# Galacto‐conjugation of Navitoclax as an efficient strategy to increase senolytic specificity and reduce platelet toxicity

**DOI:** 10.1111/acel.13142

**Published:** 2020-03-31

**Authors:** Estela González‐Gualda, Marta Pàez‐Ribes, Beatriz Lozano‐Torres, David Macias, Joseph R. Wilson, Cristina González‐López, Hui‐Ling Ou, Sofía Mirón‐Barroso, Zhenguang Zhang, Araceli Lérida‐Viso, Juan F. Blandez, Andrea Bernardos, Félix Sancenón, Miguel Rovira, Ljiljana Fruk, Carla P. Martins, Manuel Serrano, Gary J. Doherty, Ramón Martínez‐Máñez, Daniel Muñoz‐Espín

**Affiliations:** ^1^ CRUK Cambridge Centre Early Detection Programme Department of Oncology Hutchison/MRC Research Centre University of Cambridge Cambridge UK; ^2^ Instituto Interuniversitario de Investigación de Reconocimiento Molecular y Desarrollo Tecnológico (IDM) Universitat Politècnica de València Universitat de València Valencia Spain; ^3^ Unidad Mixta UPV‐CIPF de Investigación en Mecanismos de Enfermedades y Nanomedicina Centro de Investigación Príncipe Felipe Universitat Politècnica de València Valencia Spain; ^4^ CIBER de Bioingeniería, Biomateriales y Nanomedicina (CIBER‐BBN) Madrid Spain; ^5^ Unidad Mixta de Investigación en Nanomedicina y Sensores IIS La Fe Universitat Politècnica de València Valencia Spain; ^6^ Senolytic Therapeutics S.L. Parc Científic de Barcelona Barcelona Spain; ^7^ Institute for Research in Biomedicine (IRB Barcelona) The Barcelona Institute of Science and Technology (BIST), Catalan Institution for Research and Advanced Studies (ICREA) Barcelona Spain; ^8^ Department of Chemical Engineering and Biotechnology University of Cambridge Cambridge UK; ^9^ Bioscience Oncology R&D AstraZeneca Cambridge UK; ^10^ Department of Oncology Cambridge University Hospitals NHS Foundation Trust Addenbrooke's Hospital Cambridge UK

**Keywords:** cellular senescence, chemotherapy‐induced senescence, lung cancer, Navitoclax (ABT-263), prodrug, senolytics, thrombocytopenia

## Abstract

Pharmacologically active compounds with preferential cytotoxic activity for senescent cells, known as senolytics, can ameliorate or even revert pathological manifestations of senescence in numerous preclinical mouse disease models, including cancer models. However, translation of senolytic therapies to human disease is hampered by their suboptimal specificity for senescent cells and important toxicities that narrow their therapeutic windows. We have previously shown that the high levels of senescence‐associated lysosomal β‐galactosidase (SA‐β‐gal) found within senescent cells can be exploited to specifically release tracers and cytotoxic cargoes from galactose‐encapsulated nanoparticles within these cells. Here, we show that galacto‐conjugation of the BCL‐2 family inhibitor Navitoclax results in a potent senolytic prodrug (Nav‐Gal), that can be preferentially activated by SA‐β‐gal activity in a wide range of cell types. Nav‐Gal selectively induces senescent cell apoptosis and has a higher senolytic index than Navitoclax (through reduced activation in nonsenescent cells). Nav‐Gal enhances the cytotoxicity of standard senescence‐inducing chemotherapy (cisplatin) in human A549 lung cancer cells. Concomitant treatment with cisplatin and Nav‐Gal in vivo results in the eradication of senescent lung cancer cells and significantly reduces tumour growth. Importantly, galacto‐conjugation reduces Navitoclax‐induced platelet apoptosis in human and murine blood samples treated ex vivo, and thrombocytopenia at therapeutically effective concentrations in murine lung cancer models. Taken together, we provide a potentially versatile strategy for generating effective senolytic prodrugs with reduced toxicities.

## Graphical abstract

We have developed a galactose‐conjugated derivative of Navitoclax (Nav‐Gal) with a broad‐spectrum senolytic activity. We show that Nav‐Gal efficiently kills chemotherapy‐induced senescent cells in xenografts and orthotopic in vivo models of NSCLC, resulting in impaired tumour progression. Importantly, our prodrug prevents Navitoclax‐induced platelet apoptosis in human samples and murine models. In summary, we provide a potentially versatile strategy for generating effective senolytic prodrugs with reduced toxicities.

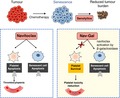

## 1 INTRODUCTION

Senescence is an evolutionarily conserved cellular response to severe stress and damage characterized by stable cell cycle arrest, upregulation of pro‐survival signalling pathways and the induction of a complex secretory phenotype, termed the senescence‐associated secretory phenotype (SASP) (Gorgoulis et al., 2019). Physiological roles of cellular senescence include prevention of the propagation of damaged or dysfunctional cells and promotion of tissue repair; these processes are facilitated by immune system cells driving the clearance of senescent cells (Muñoz‐Espín & Serrano, 2014). Senescence plays an active role in tumour suppression, reprogramming, tissue regeneration, wound healing and embryogenesis. However, the processes of senescence and immune clearance are dysregulated during aging, where senescent cells accumulate in tissues contributing to the onset and progression of multiple age‐related disorders through a variety of cell autonomous and paracrine effects that disrupt tissue homeostasis (Muñoz‐Espín & Serrano, 2014). Among other pathologies, senescence is associated with fibrotic disorders, cardiovascular diseases, obesity, diabetes, osteoarthritis, neurological disorders, inflammatory diseases and cancer (Childs et al., 2017; McHugh & Gil, 2018).

Conclusive evidence is accumulating that therapy‐induced senescent cells (both with a cancerous and stromal origin) can drive tumorigenesis, either by releasing complex pro‐inflammatory and tumour‐promoting SASP cocktails (Faget, Ren, & Stewart, 2019; Gonzalez‐Meljem, Apps, Fraser, & Martinez‐Barbera, 2018), or by the reversion of the cell cycle arrest and the acquisition of stemness and aggressive clonogenic growth potentials (Lee & Schmitt, 2019). Genotoxic stress, which is induced by many chemotherapies, promotes cellular senescence. Remarkably, it has been shown that chemotherapy‐induced senescent breast cancer cells can promote tumour relapse in the lung (Demaria et al., 2017), and senescent stromal cells form the niche that promotes metastasis in the bone (Luo et al., 2016). In the case of NSCLC, neoadjuvant platinum‐based chemotherapy results in the accumulation of senescent cancer cells in patients and evidence has been found of the escape of replicative arrest in humans (therapy‐induced) senescent lung cancer cells (Roberson, Kussick, Vallieres, Chen, & Wu, 2005). Therefore, treatment modalities that eliminate therapy‐induced senescent cells may be critical for tumour eradication.

Recent research has identified targetable vulnerabilities of senescent cells that can be exploited by a novel group of drugs called senolytics. These compounds preferentially kill senescent cells by different mechanisms (Lozano‐Torres et al., 2019; Paez‐Ribes, González‐Gualda, Doherty, & Muñoz‐Espín, 2019). Senolytics include the BCL‐2 family inhibitors Navitoclax (ABT‐263) (Zhu et al., 2016) and ABT‐737 (Yosef et al., 2016); the flavonoid fisetin (Yousefzadeh et al., 2018); combinations of tyrosine kinase inhibitors and flavonoids (e.g. dasatinib and quercetin; Zhu et al., 2015); FOXO4‐p53 interfering peptides (Baar et al., 2017); HSP90 chaperone inhibitors (Fuhrmann‐Stroissnigg et al., 2017); and other compounds such as piperlongumine (Wang et al., 2016) and cardiac glycosides (Guerrero et al., 2019; Triana‐Martínez et al., 2019). Senolytics have emerged as promising agents for treatment of pulmonary fibrosis, atherosclerosis, osteoarthritis, type 1 and 2 diabetes mellitus, and neurocognitive decline. They can also rejuvenate aged hematopoietic and muscle stem cells and extend the lifespan of naturally aged mice (Paez‐Ribes et al., 2019).

Despite successful preclinical *proofs‐of‐concept* for senolytics, their potential translatability is hampered by their associated toxicities, necessitating the development of more specific, and less toxic, second‐generation senolytics. Navitoclax has been validated in a variety of preclinical models showing high potency in killing senescent cells—however, it also has significant on‐target haematological toxicity, including thrombocytopenia (Cang, Iragavarapu, Savooji, Song, & Liu, 2015). This narrows its therapeutic window and can preclude concomitant treatment with other agents with haematological toxicities. While targetable vulnerabilities of senescence have been discovered, these are often also present in nonsenescent tissues leading to problems with specifically targeting senescent cells. One consistent feature of senescent cells is their enrichment in lysosomes and lysosomal proteins, including senescence‐associated β‐galactosidase (SA‐β‐gal) which is widely used as a marker of senescence (Hernandez‐Segura, Nehme, & Demaria, 2018) and can be readily detected (Dimri et al., 1995). We previously showed that the encapsulation of nanoparticles with galacto‐oligosaccharides (GalNPs) is an efficient method to preferentially deliver cytotoxic drugs and tracers to the lysosomes of senescent cells where SA‐β‐gal activity digests the galacto‐oligosaccharides, thereby releasing the cargo (Agostini et al., 2012; Muñoz‐Espín et al., 2018). We demonstrated that galacto‐encapsulated doxorubicin is preferentially released into fibrotic tissues and tumours accumulating senescent cells, and its concomitant administration with the senescence‐inducing anti‐cancer treatment palbociclib effectively halts tumour growth in xenograft models of melanoma and non‐small‐cell lung cancer (NSCLC) (Muñoz‐Espín et al., 2018). We have also shown that a fluorescent probe covalently linked to multi‐acetylated galactose is preferentially digested by senescent cells, releasing the free fluorophore (Lozano‐Torres et al., 2017). The presence of multiple acetyl moieties in the galactose residue is thought to render it membrane‐permeable and therefore accessible to the lysosomal compartment (Lee et al., 2019).

**FIGURE 1:**
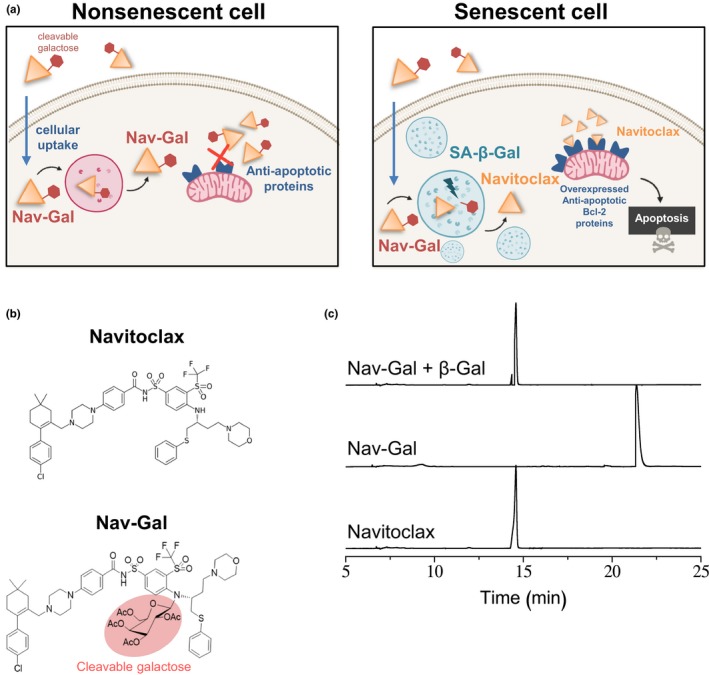
Galacto‐conjugation of the senolytic Navitoclax into a new generation senolytic prodrug, namely Nav‐Gal, as an efficient strategy for selective senolysis. (a) Schematic representation of the mechanism of action of Nav‐Gal prodrug. Nav‐Gal is passively taken up by both nonsenescent and senescent cells. In nonsenescent cells, its conjugation with a cleavable galactose renders it inactive and unable to inhibit anti‐apoptotic proteins, such as BCL‐2, preventing the induction of apoptosis. In senescent cells, the increased lysosomal and galactosidase activity, a hallmark of cellular senescence, allows the hydrolysis of the cleavable galactose, resulting in the release of active Navitoclax into the cytoplasm of senescent cells. Free Navitoclax will inhibit anti‐apoptotic BCL‐2 proteins, which are overexpressed in senescent cells, driving specific apoptosis of these cells. (b) Chemical structures of Nav‐Gal prodrug and Navitoclax. The presence of galactopyranoside, covalently linked to the N of bis(sulfonyl)aniline as synthesized in this prodrug, hinders two key interactions: (i) π‐π interaction between the phenylthioether moiety and the bis(sulfonyl)aniline ring; and (ii) the hydrogen bond between morpholine with Tyr‐199 (Liu, Zhang, Huang, Tan, & Zhang, 2018), thereby preventing the inhibitory effect of the molecule. This moiety, the galactopyranoside, can be hydrolyzed in the presence of β‐galactosidase (cleavable galactose). (c) Chromatograms depicting hydrolysis reaction of Nav‐Gal aqueous solutions in the presence of human β‐galactosidase followed by HPLC‐UV as described in the text

Here, we have modified Navitoclax with an acetylated galactose to exploit the enriched SA‐β‐gal activity of senescent cells (Figure 1a). Using a variety of model systems, we show that galacto‐conjugation of Navitoclax, which we name Nav‐Gal, results in a prodrug with selective, pro‐apoptotic senolytic activity released in senescent cells that is dependent on GLB1 activity. Concomitant treatment of Nav‐Gal with the senescence‐inducing chemotherapy cisplatin (CDDP) efficiently arrests tumour progression in models of orthotopically transplanted murine lung adenocarcinoma cells, and in a tumour xenograft model of human NSCLC. Importantly, galacto‐conjugation of Navitoclax reduces thrombocytopenia in treated mice at therapeutically effective doses, as well as apoptosis of platelets in human blood samples treated ex vivo. Overall, we propose galacto‐conjugation of cytotoxic drugs as a versatile methodology for developing second‐generation prodrugs with high senolytic activity and reduced toxicity. We provide evidence of the efficacy of combining senescence‐inducing chemotherapies with senotherapies in cancer, with potential for clinical application.

## 2 RESULTS

### 2.1 Synthesis, characterization and hydrolysis reaction studies

RNA interference and drug screening approaches led to the identification of the anti‐apoptotic BCL‐2 protein family as potential therapeutic targets in senescent cells, and Navitoclax (ABT‐263) as a drug with a potent senolytic activity (Chang et al., 2016; Zhu et al., 2016). Here, we have engineered a novel prodrug, namely Nav‐Gal, by modifying the molecular structure of Navitoclax following the synthetic procedure shown in Figure S1A. To do so, Navitoclax was reacted with 2,3,4,6‐tetra‐O‐acetyl‐α‐D‐galactopyranosyl bromide (Gal) through bimolecular nucleophilic substitution (S_N2_) in the presence of potassium carbonate yielding the Nav‐Gal prodrug (Figure 1b), which was comprehensively characterized by several nuclear magnetic resonance techniques (NMR), including ^1^H‐NMR, ^13^C‐NMR and correlated spectroscopy (COSY) NMR, as well as by attenuated total reflectance (ATR) and high‐resolution mass spectrometry (HRMS) (Figures S1B–F). The ^1^H NMR signal centred at 5.6 ppm (Figure S1B) corresponds to the anomeric proton of the attached galactose and indicates the successful covalent linkage of the monosaccharide to Navitoclax. In addition, the appearance of a signal centred at 1,749 cm^−1^ in the ATR spectra (Figure S1E) indicates that galactose remains acetylated after purification. Correct functionalization to the nitrogen atom of bis(sulfonyl)aniline is further corroborated through the fragmentation peaks observed in the mass spectrum (Figure S1F). After extensive structural characterization, we examined the ability of Nav‐Gal to be enzymatically hydrolyzed to Navitoclax. GLB1 is the human lysosomal β‐galactosidase responsible for the SA‐β‐gal activity (Dimri et al., 1995). We used high performance liquid chromatography (HPLC) to examine the time‐dependent hydrolysis reaction of PBS (pH 7)‐DMSO (0.01%) solutions containing Nav‐Gal in the presence of recombinant GLB1 and compared the peaks with those of Navitoclax (Figure 1c). The obtained chromatograms showed the Nav‐Gal peak with the subsequent appearance of free Navitoclax signal in the presence of the β‐galactosidase activity of the lysosomal enzyme and confirmed that Nav‐Gal was completely hydrolyzed. No significant spontaneous Nav‐Gal hydrolysis was observed (data not shown).

### 2.2 Nav‐Gal is a prodrug with effective wide‐ranging senolytic activity that depends on GLB1 activity

**FIGURE 2:**
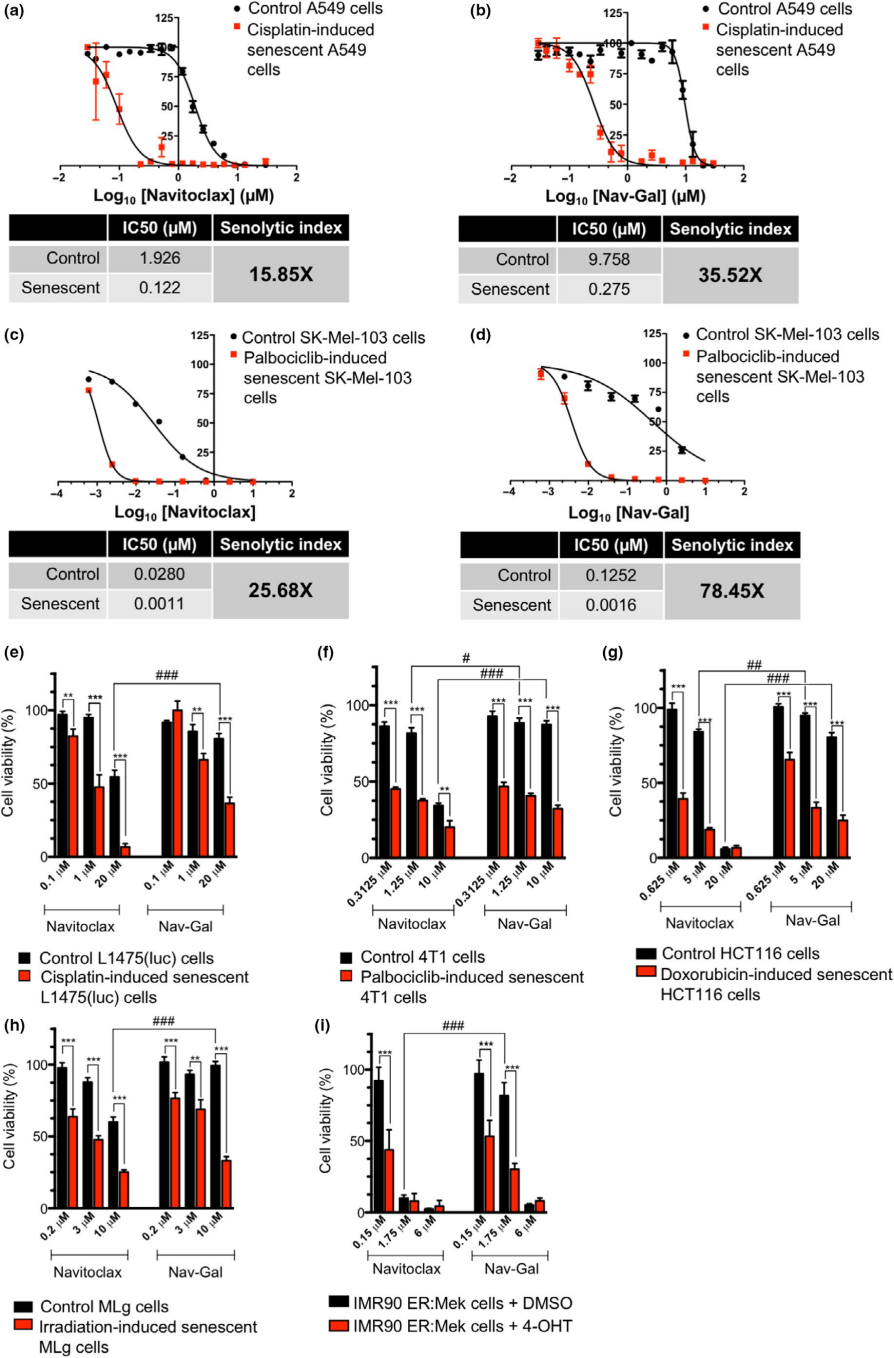
The prodrug Nav‐Gal shows efficient, broad range, senolytic activity and an increased senolytic index, conferring a protective effect on nonsenescent cells. (a, b) Quantification of viability and half maximal inhibitory concentration (IC50) of (a) Navitoclax and (b) Nav‐Gal on control and cisplatin‐induced senescent A549 cells. Senolytic indices for each drug are shown in the tables below. Viability assay on A549 cells was performed as *n* = 5, and graphs depict one representative repeat. (c, d) Quantification of viability and maximal inhibitory concentration (IC50) of (c) Navitoclax and (d) Nav‐Gal on control and palbociclib‐induced senescent SK‐Mel‐103 cells. Senolytic indices of each drug are shown in the tables below. Viability assay on SK‐Mel‐103 cells was performed as *n* = 3, and graphs depict one representative repeat. (e–i) Quantification of cell viability upon Navitoclax and Nav‐Gal treatment of control and (e) cisplatin‐induced senescent *KRas* ^G12D/WT^;*p53* ^−/−^ lung adenocarcinoma tumour cells L1475(luc) (*n* = 4), (f) palbociclib‐induced senescent 4T1 cells (*n* = 3), (g) doxorubicin‐induced senescent HCTT116 cells (*n* = 3), (h) irradiation‐induced senescent MLg cells (*n* = 3) and (i) oncogene‐induced senescent IMR90 cells (previously treated for 72 hr with 200 nM 4‐hydroxytamoxifen (4‐OHT)) (n=3). Data in (e‐i) represent mean ± *SD* of replicates, and statistical significance was calculated using two‐way ANOVA ; *p < .05, **p < .01, ***p < .001; #*p* < .05, ##*p* < .01, ###*p* < .001

Since senescent cells are commonly characterized by high lysosomal SA‐β‐gal activity, we hypothesized that galacto‐conjugated Navitoclax would be preferentially processed and activated in senescent cells and hence could function as a prodrug with more selective senolytic activity. To investigate this, we performed cell viability assays using a model of therapy‐induced senescence. The lung carcinoma cell line A549 was treated with cisplatin (CDDP) for 10 days, resulting in elevated SA‐β‐gal activity and expression of markers of senescence (Figure S2A–C). Cisplatin‐treated A549 cells were then subjected to increasing doses of either Navitoclax or Nav‐Gal for 72 hr. As shown in Figure 2a, the concentration of Navitoclax required to induce death in 50% of the cells, termed half maximal inhibitory concentration (IC50), after 72 hr of treatment was 1.926 µM for nonsenescent and 0.122 µM for cisplatin‐induced senescent A549 cells, while the IC50 for Nav‐Gal was 9.758 µM for nonsenescent and 0.275 µM for senescent A549 cells (Figure 2b). A similar approach was performed in a model of palbociclib‐induced senescence with a human melanoma cell line (SK‐Mel‐103) (Figure 2c,d and Figure S2A‐C). These results show (in two different models of chemotherapy‐induced senescence) that Nav‐Gal has an improved senolytic index over Navitoclax and indicates that this effect is mainly mediated by a higher degree of protection of nonsenescent cells from cytotoxic activity. In an attempt to determine whether senolytic activity and nonsenescent cell protection by Nav‐Gal were more widely observed, we then performed cell viability assays using a variety of cell lines and diverse triggers of cellular senescence. Figure 2e‐i show the effects on cell viability of both Navitoclax and Nav‐Gal at different concentrations in cisplatin‐induced senescent mouse lung adenocarcinoma (*Kras^G12D/+^;p53^−/−^* (KP) L1475(luc) cells) (Turrell et al., 2017), palbociclib‐induced senescent mouse breast cancer 4T1 cells, doxorubicin‐induced senescent human colorectal carcinoma HCT116 cells, irradiation‐induced senescent mouse lung fibroblasts (MLg) and oncogene‐induced senescent human lung fibroblasts (IMR90), versus their nonsenescent counterparts. Efficient implementation of cellular senescence using these triggers was confirmed by SA‐β‐gal staining and Western blotting for senescence markers (Figure S2A‐C). In all the human/murine cell types tested, and independently of the senescence‐inducing trigger used, Nav‐Gal showed effective senolytic activity and a significantly higher degree of protection of nonsenescent cells when compared to Navitoclax.

**FIGURE 3:**
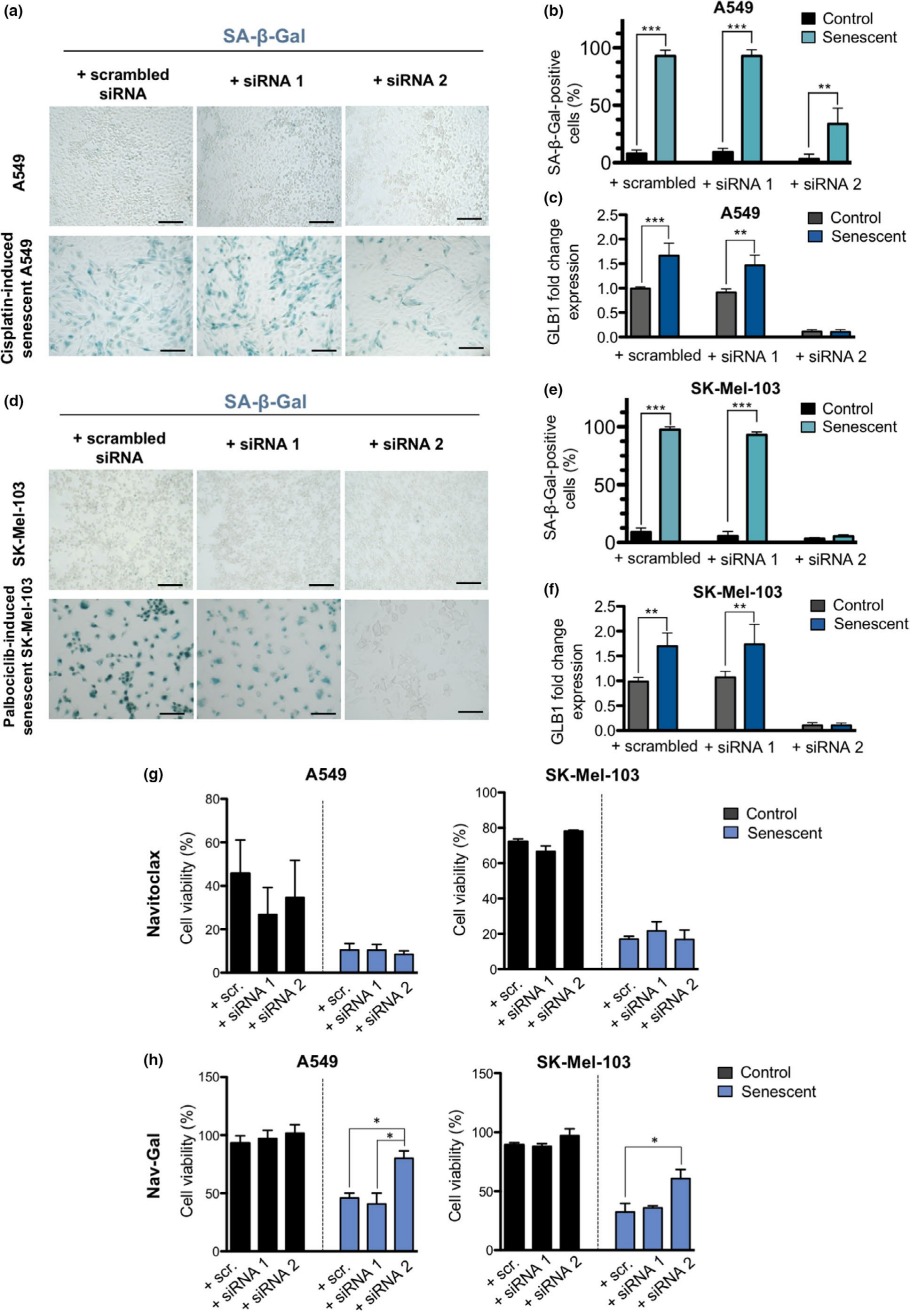
*GLB1* transient downregulation prevents the senolytic activity of Nav‐Gal. (a) Representative images of SA‐β‐gal staining of control and cisplatin‐induced senescent A549 cells 48 hr after transfection with scrambled siRNA, siRNA 1 and siRNA 2. Scale bar = 200 μm. (b) Percentage of SA‐β‐gal‐positive cells in conditions presented in (a). Bars represent mean ± *SD* (*n* = 3). (c) *GLB1* fold change gene expression in control and senescent A549 cells 48 hr post‐transfection with different siRNAs. (d) Representative images of SA‐β‐gal staining of control and palbociclib‐induced senescent SK‐Mel‐103 cells 48 hr after transfection with scrambled siRNA, siRNA 1 and siRNA 2. Scale bar = 200 μm. (e) Percentage of SA‐β‐gal‐positive cells in conditions presented in (d). Bars represent mean ± *SD* (*n* = 3). (f) *GLB1* fold change gene expression in control and senescent SK‐Mel‐103 cells 48 hr post‐transfection with different siRNAs. (g) Quantification of cell viability upon 48 hr Navitoclax treatment of control and cisplatin‐induced senescent A549 cells (10 μM Navitoclax) (left) and control and palbociclib‐induced senescent SK‐Mel103 cells (7.5 μM Navitoclax) (right) previously transfected with different experimental siRNAs against *GLB1* transcript. (h) Quantification of cell viability upon 48 hr Nav‐Gal treatment of control and cisplatin‐induced senescent A549 cells (10 μM Nav‐Gal) (left) and control and palbociclib‐induced senescent SK‐Mel103 cells (7.5 μM Nav‐Gal) (right) previously transfected with different experimental siRNAs against *GLB1* transcript. Note that siRNA 1 was not functional in all the experiments and hence used as an internal negative control. All bars represent mean ± *SEM* (*n* = 3). One‐way ANOVA followed by Tukey's post‐tests were performed to calculate the significance of the results; *p < .05, **p < .01, ***p < .001

To determine whether the senolytic effect of the prodrug Nav‐Gal depends on the increased lysosomal β‐galactosidase activity of senescent cells, siRNAs were used to knock‐down the expression of *GLB1* in A549 and SK‐Mel‐103 cells. As shown in Figure 3a‐f, siRNA2 efficiently downregulated the transcription of *GLB1* at 48 hr post‐transfection and resulted in a significantly decreased number of SA‐β‐galactosidase‐positive cells in both cell lines, when compared to scrambled siRNA and siRNA 1. While the killing efficiency of Navitoclax was maintained in the senescent cells in both cell lines (Figure 3g), transient downregulation of *GLB1* significantly prevented the senolytic activity of Nav‐Gal (Figure 3h) in A549 and SK‐Mel‐103 cells, indicating that the selective senolysis of the prodrug is (at least) partially driven by the increased *GLB1* expression of senescent cells.

### 2.3 Nav‐Gal induces apoptosis of senescent cells while preserving viability of nonsenescent cells

**FIGURE 4:**
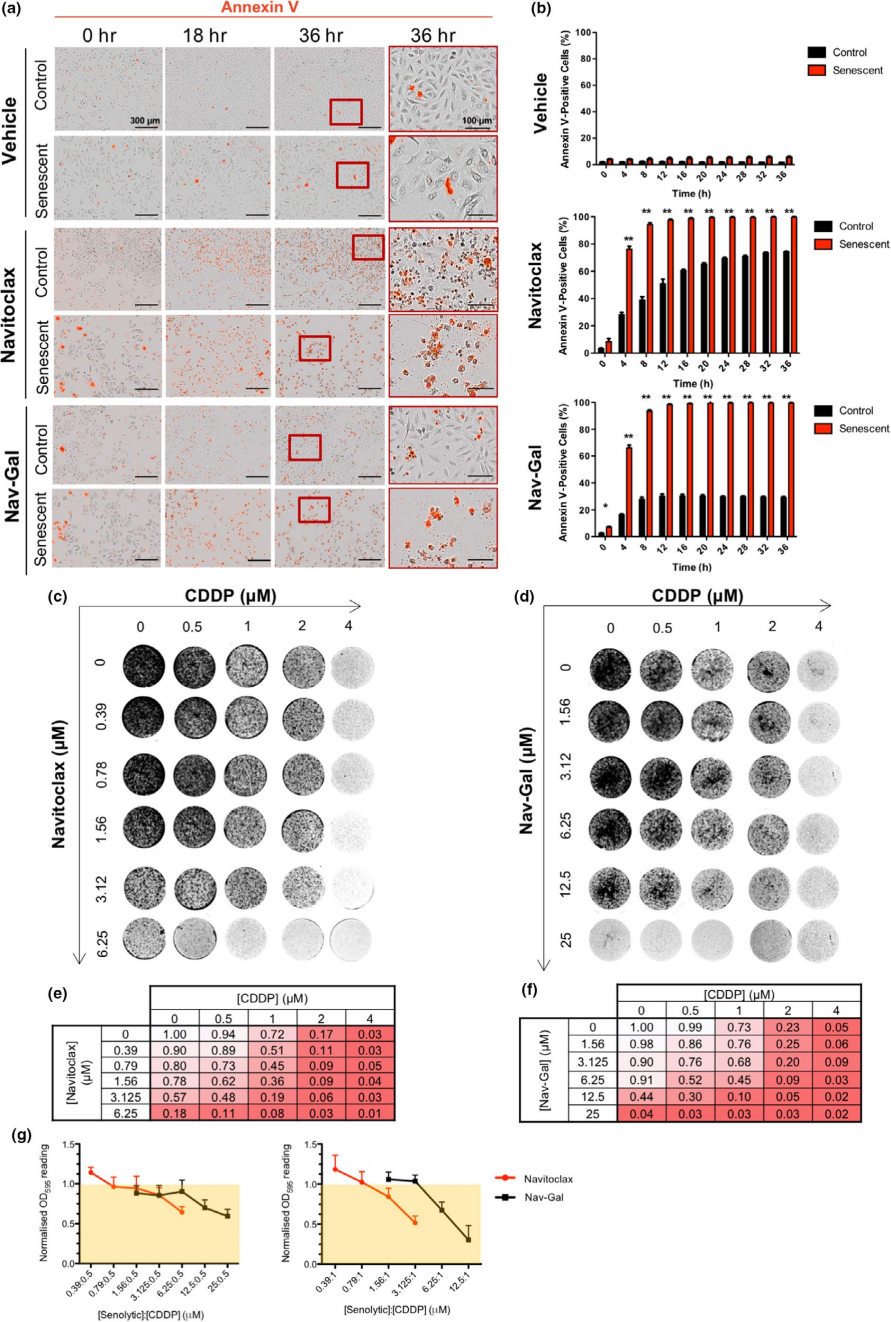
The galacto‐conjugated prodrug Nav‐Gal shows an enhanced effect when combined to senescence‐inducing cisplatin treatment and a lower induction of apoptosis of nonsenescent cells. (a) Representative images of cell viability showing staining for annexin V (red) of control or cisplatin‐induced senescent A549 cells, exposed to vehicle, Navitoclax (10 μM) or Nav‐Gal (10 μM) treatment over time. Red scale bar = 300 μm; black scale bar = 100 μm. (b) Average percentage of annexin V‐positive cells in control and cisplatin‐induced senescent A549 cells exposed to vehicle (top), Navitoclax (10 μM; middle) or Nav‐Gal (10 μM; bottom) treatment over time. Data represent mean ± *SD* (*n* = 3), where for each biological repeat the percentage of annexin V‐positive cells was calculated in 3 independent technical repeats per experimental condition. Statistical significance was calculated using two‐tailed Student's *t* tests; *p < .05, **p < .001. (c, d) Representative images of clonogenic survival of A549 cells exposed to increasing concomitant concentrations of cisplatin (CDDP) and (c) Navitoclax or (d) Nav‐Gal as specified in axis. (e, f) Numerical heat map representation of normalized mean clonogenic potential after 10 days of increasing concomitant treatment with CDDP and (e) Navitoclax or (f) Nav‐Gal, where 1 = maximum clonogenic potential corresponding to untreated condition (*n* = 3). (g) Co‐coefficient of drug interaction (CDI) value trend of Navitoclax and Nav‐Gal across 0.5 μM cisplatin treatment (left) and 1 μM cisplatin treatment (right). CDI < 1 (yellow area) indicates a synergistic effect, where CDI values closer to 0 correlate to higher synergy between the drugs concomitantly used. Data represent mean ± *SD* (*n* = 3)

The use of Navitoclax has been described to induce apoptosis preferentially in senescent cells through inhibition of the BCL‐2‐regulated pathway (Zhu et al., 2016). To assess whether Nav‐Gal and Navitoclax killed senescent cells by the same end mechanism, cisplatin‐induced senescent lung cancer A549 cells and palbociclib‐induced senescent melanoma SK‐Mel‐103 cells were treated with Navitoclax or Nav‐Gal (or vehicle) in parallel, with automated real‐time measurement of apoptosis study using a live‐cell analysis system. Figure 4a,b and Figure S3A–D show that treatment with either Navitoclax or Nav‐Gal induced apoptosis preferentially in chemotherapy‐induced senescent cells (as inferred from an increased annexin V signal). Importantly, and consistent with the results above (Figure 2a,b), the induction of apoptosis was lower in control nonsenescent cells treated with Nav‐Gal compared with Navitoclax (both at a high dose; 10 μM), particularly at later time points. While over 70% of nonsenescent A549 cells presented a strong signal for annexin V after 36 hr of treatment with Navitoclax, this was true for only ~30% of the cells after treatment with the same dose of Nav‐Gal (Figure 4a,b and Figure S3A). Consistent effects were observed with the melanoma cell line SK‐Mel‐103 (Figure S3B–D). These results indicate that Nav‐Gal kills senescent cells by inducing apoptosis and that it protects against nonsenescent cell death to a higher extent than Navitoclax.

### 2.4 Cisplatin and Nav‐Gal have additive anti‐tumour effects when used concurrently and sequentially

Effective anti‐cancer treatments have enhanced clinically beneficial effects when given in combination, including in NSCLC. We therefore next sought to ascertain the efficacy of concurrent cisplatin and Nav‐Gal treatment in vitro. We first used clonogenic assays to determine the efficacy of the combination of senescence‐inducing cisplatin and Navitoclax, Nav‐Gal or vehicle, administered concomitantly to A549 cells. Compared with monotherapies, the combination of cisplatin and a senolytic drug was substantially more effective at inhibiting cell proliferation (Figure 4c‐f). From 0 to 2 µM cisplatin, as the concentration of both Navitoclax and Nav‐Gal increases, additional impairment of cell growth was observed. There was also a significant reduction in the concentration of Nav‐Gal required to produce growth inhibition in 1 µM cisplatin versus 0 µM cisplatin (Figure S3G). Similar to the IC50 experiments (Figure 2a,b), a higher dose of Nav‐Gal than Navitoclax was required to achieve the same effect, likely due to the requirement for Nav‐Gal processing and activation by lysosomal β‐galactosidase activity. While both senolytic drugs showed an additive effect in combination with cisplatin, increasing doses of Nav‐Gal exhibited coefficients of drug interactions (CDIs) <1 and closer to 0 in combination with 1 μM cisplatin (Figure 4g), which suggests that the combination of drugs may be synergistic (Zhao et al., 2014). Of note, these observations were also validated in the context of a sequential treatment, where colonies were first exposed to increasing concentrations of cisplatin for 7 days, followed by 7 days of senotherapy (Figure S3E,F).

### 2.5 Therapeutic activity of Nav‐Gal in xenografts and orthotopic models of NSCLC

**FIGURE 5:**
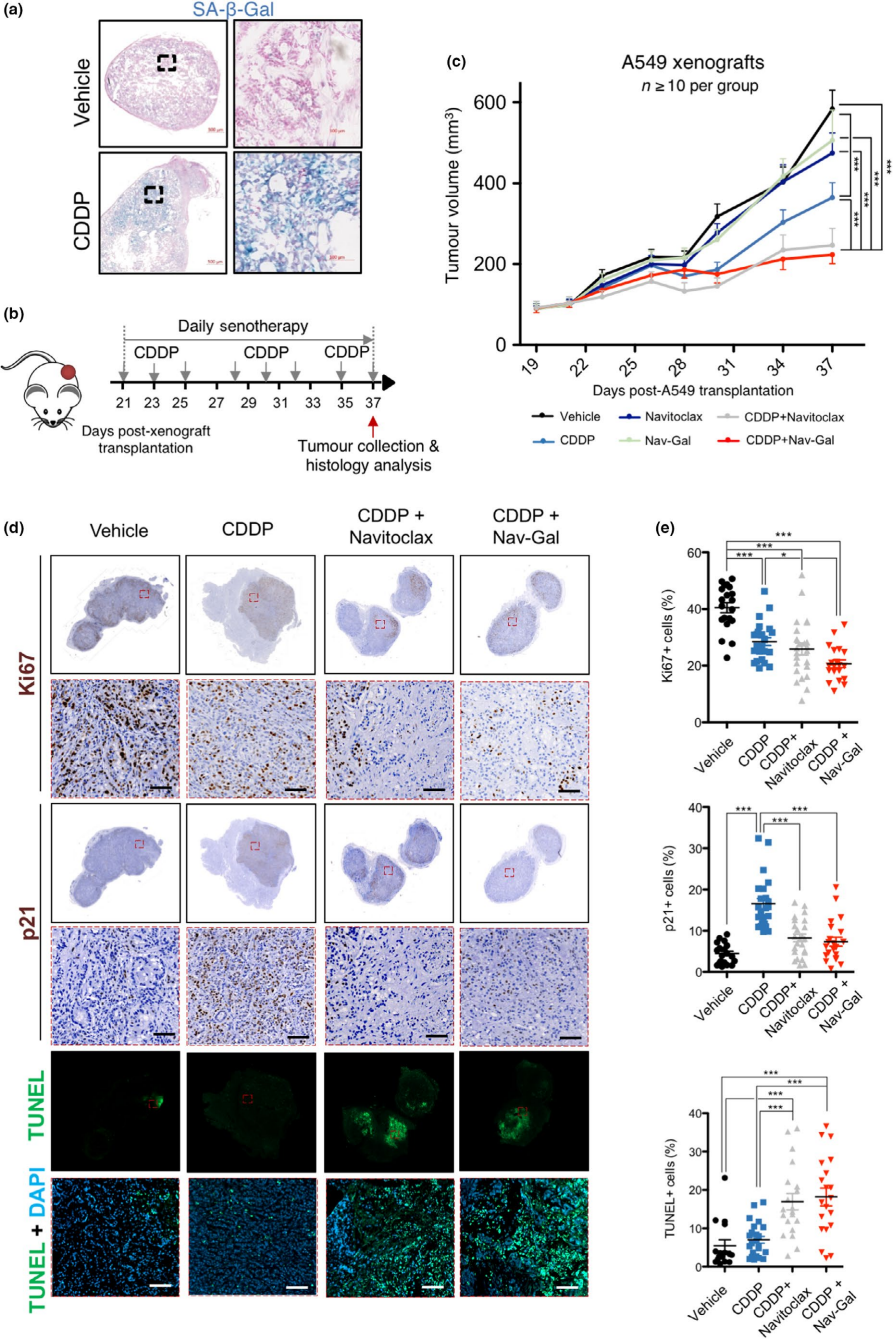
Concomitant treatment with the prodrug Nav‐Gal and cisplatin significantly inhibits tumour growth in a human lung cancer xenograft mouse model. (a) Representative images of A549 xenografts stained for SA‐β‐Gal activity (in blue) after treatment with cisplatin or vehicle. (b) Schematic representation of concomitant treatment on A549 xenograft‐bearing mice. (c) Tumour volume of A549 xenografts in mice concomitantly treated with cisplatin and Navitoclax or Nav‐Gal (as described in (b); *n* ≥ 10 tumours per group. Data represent mean ± *SEM*. (d) Representative histological images of tumours at the end of concomitant treatment, stained for Ki67 and p21 expression, and labelled using TUNEL staining. Scale bar = 100 μm. (e) Percentage of Ki67‐ (top), p21‐ (middle) and TUNEL‐positive (bottom) cells in tumours from animals treated with vehicle, cisplatin, or cisplatin and Navitoclax or Nav‐Gal concomitantly (*n* ≥ 5 tumours per group). For quantification, a total of 4 fields per tumour was analyzed, covering most of the total tumour area. Two‐way ANOVA followed by Bonferroni post‐tests was performed to calculate the significance of the results; *p < .05; **p < .01; ***p < .001

In order to validate the efficiency of the prodrug Nav‐Gal in combination with senescence‐inducing chemotherapy in vivo, we used a model whereby A549 cells were transplanted subcutaneously into the flanks of severe combined immunodeficient (SCID) mice. In a first approach, we aimed to assess the induction of senescence in tumour‐bearing mice treated with cisplatin. Histological analyses of the tumours collected after treatment showed evidence of senescence induction upon cisplatin administration (as inferred from SA‐β‐gal positivity) (Figure 5a). The effects of concomitant treatment with cisplatin and each senolytic were then investigated. Once tumours reached an average volume of 100 mm^3^, mice were treated with cisplatin and daily doses of Navitoclax and Nav‐Gal, alone or in combination, for 17 days (as shown in Figure 5b). Importantly, both drugs significantly improved the tumour growth inhibition of cisplatin (Figure 5c). The combination of cisplatin with either Navitoclax or Nav‐Gal had comparable effects on tumour growth inhibition, but both showed statistically significant differences with control and monotherapy groups. In contrast, Navitoclax and Nav‐Gal had no appreciable effect on tumour growth when administered in the absence of cisplatin, indicating that their therapeutic activities require concomitant induction of senescence by cisplatin in this model system. Consistently, histological analyses of the tumours revealed that cisplatin treatment results in increased p21 positivity (correlating with Western blot analyses of cisplatin‐treated cells in vitro, Figure S2C) and decreased Ki67 positivity, which together constitute a hallmark of senescent cells (Figure 5d,e). Treatment with cisplatin and either Navitoclax or Nav‐Gal exhibited reduced levels of p21 and Ki67 positivity, alongside a strong TUNEL signal, strongly suggesting that apoptosis of senescent cells facilitates the anti‐tumour effect. Additionally, a therapeutic effect of both Navitoclax and Nav‐Gal in tumour growth was observed in a sequential treatment after one week of cisplatin administration, suggesting a potential application as adjuvant therapy for clearing senescent cells (Figure S4A,B).

To obtain further evidence of the therapeutic potential of Nav‐Gal in combination with senescence‐inducing chemotherapy in a more physiological context, we used an orthotopic model of NSCLC. Here, wild‐type C57BL/6J mice were transplanted (via tail vein injection) with a syngeneic luciferase‐expressing KP lung adenocarcinoma cell line (L1475luc) (Turrell et al., 2017). Once tumours were established in the lungs, baseline luciferase signals were obtained and mice were then treated for 10 days with cisplatin alone, cisplatin and Nav‐Gal in combination, or their vehicles in combination, using the experimental scheme shown in Figure S5A. Representative images of the luciferase signal at initial‐ and end‐points are shown in Figure S5B. Luciferase signal monitoring demonstrated that concomitant treatment of the mice with cisplatin and Nav‐Gal significantly decreased tumour burden (Figure S5C) compared with cisplatin monotherapy. Histological analysis of the lungs showed high burden of senescent cells only in cisplatin‐treated mice (as evidenced by increased SA‐β‐gal and p21 levels), and decreased SA‐β‐gal and p21 levels and higher TUNEL staining in mice concomitantly treated with cisplatin and Nav‐Gal, indicating enhanced apoptosis of senescent cells (representative areas are shown in Figure S5D).

Taken together, these results demonstrate that the combination of senescence‐inducing therapy with senotherapy is highly effective in inhibiting tumour growth in vivo, providing preclinical *proof‐of‐principle* of the therapeutic benefits of using Nav‐Gal as a potent prodrug with senolytic activity.

### 2.6 Nav‐Gal has reduced platelet toxicity when compared with Navitoclax

**FIGURE 6:**
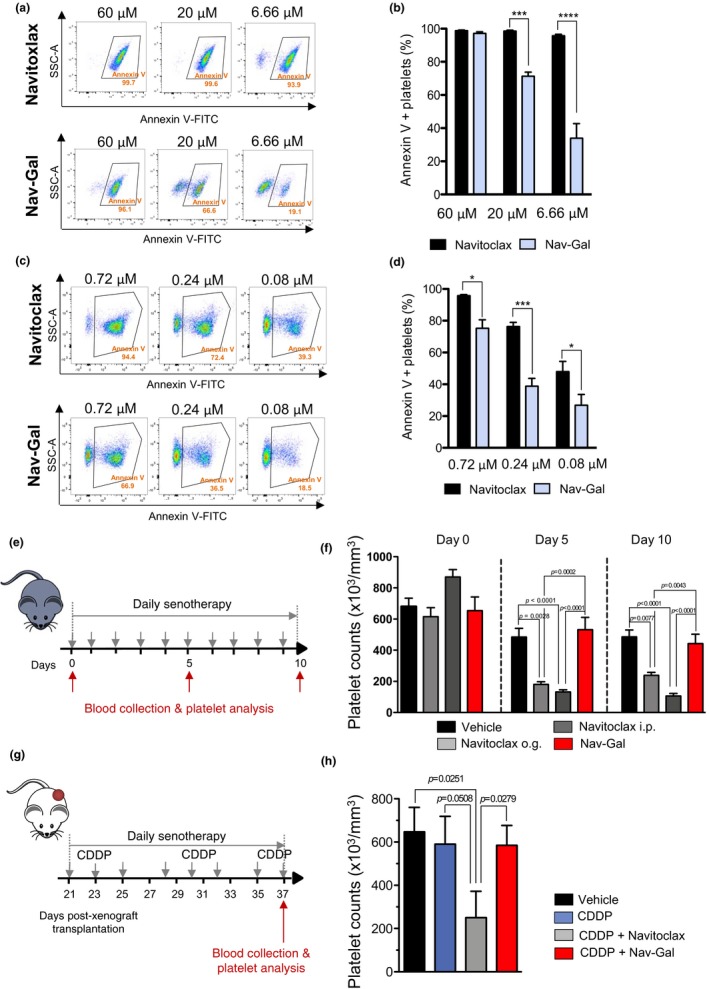
Nav‐Gal reduces platelet apoptosis in human and mouse blood ex vivo, and prevents thrombocytopenia in mice treated concomitantly with chemotherapy, compared to Navitoclax. (a) Blood from healthy human volunteers was collected and treated ex vivo with 60, 20 and 6.66 μM Navitoclax or Nav‐Gal as described. Apoptosis was analyzed after annexin V‐FITC antibody incubation by flow cytometry. Graphs show proportion of apoptotic platelets upon each treatment based on scatter signals and annexin V expression signal after gating for CD41‐positive cells. (b) Percentage of annexin V‐positive platelets in human blood after treatment with 60, 20 and 6.66 μM Navitoclax or Nav‐Gal. Bars represent mean ± *SD* (*n* = 3). (c) Blood from wild‐type C57BL/6J mice was collected and treated ex vivo with 0.72, 0.24 and 0.08 μM Navitoclax or Nav‐Gal as described. Apoptosis was analyzed after annexin V‐FITC antibody incubation by flow cytometry. Graphs show proportion of apoptotic platelets upon each treatment based on scatter signals and annexin V expression signal after gating for CD41‐positive cells. (d) Percentage of annexin V‐positive platelets in mouse blood after treatment with 0.72, 0.24 and 0.08 μM Navitoclax or Nav‐Gal. Bars represent mean ± *SD* (*n* = 5). (e) Wild‐type C57BL/6J mice were treated daily with Navitoclax by oral gavage (o.g.) (100 mg/kg body weight), with Navitoclax administered by i.p. injection (85 mg/kg body weight) or with Nav‐Gal by o.g administration (85 mg/kg body weight) for 10 consecutive days. Blood was collected on day 0, 5 and 10 by superficial vessel puncture and platelet count was analyzed. (f) Platelet count on day 0, 5 and 10 during the treatment of wild‐type C57BL/6J mice in each experimental condition as described in (e) (vehicle, *n* = 4; Navitoclax o.g., *n* = 9; Navitoclax i.p., *n* = 8; Nav‐Gal o.g., *n* = 8). Bars represent mean ± *SEM*. (g) SCID mice bearing A549‐derived xenografts were treated with cisplatin (CDDP, 1.5 mg/kg body weight three times a week) and concomitant daily senotherapy (Navitoclax [100 mg/kg body weight] or Nav‐Gal [85 mg/kg body weight]) as shown in this schematic representation. Blood was collected after treatment by cardiac puncture and, platelet count was analyzed. (h) Platelet count in each experimental condition described in (g) upon end of treatment in vivo (vehicle and CDDP, *n* = 5; CDDP + Navitoclax, *n* = 4; CDDP + Nav‐Gal, *n* = 6). Data represent mean ± *SEM*. Two‐way ANOVA followed by Bonferroni post‐tests or one‐tailed *t* tests was performed to calculate the significance of the results; * *p* < .05; ***p < .001; ****p < .0001

Thrombocytopenia is a major dose‐limiting, and clinically important, toxicity that is produced by Navitoclax in human patients (Cang et al., 2015; Wilson et al., 2010). A potential benefit for galacto‐conjugation of senolytic drugs is the reduction of their associated toxicities, and the potential widening of their therapeutic windows. To determine whether galacto‐conjugation affected platelet toxicity, we performed ex vivo experiments with both human and murine blood samples where we exposed the whole blood to increasing concentrations of either Navitoclax or Nav‐Gal, using fluorescein‐labelled annexin V to identify apoptotic platelets by flow cytometry ((Vogler et al., 2011); Figure S6A,B). As observed in Figure 6a,b for human blood and in Figure 6c,d for mouse blood, when samples were exposed to the same concentrations of Navitoclax and Nav‐Gal, the proportion of platelets with annexin V signal was significantly higher after exposure to Navitoclax than with Nav‐Gal. This effect persists in both models, despite a significant difference in platelet sensitivity to BCL‐2 family inhibition between each model.

To determine whether Nav‐Gal also protected platelet levels at therapeutic doses in vivo, we first examined the platelet count of wild‐type C57BL/6J treated daily with senotherapy for a total of 10 days (Figure 6e). As shown in Figure 6f, daily administration of Navitoclax resulted in severe thrombocytopenia after only 5 days of treatment, independently of the route of administration. Remarkably, Nav‐Gal treatment did not cause thrombocytopenia in the mice and led to platelet count levels comparable to those of vehicle‐treated individuals, showing reduced platelet toxicity compared to Navitoclax. In order to validate our findings in the context of an in vivo cancer model, the platelet count was also assessed at end‐points of the concomitant treatment of xenograft‐bearing mice described in Figure 5b‐d. Notably, as shown in Figure 6g,h, no significant additional thrombocytopenia was observed in the group treated with cisplatin and Nav‐Gal (cf. Navitoclax). We also found that Nav‐Gal reduced platelet toxicity, either in combination with cisplatin or as monotherapy, when administered in a sequential manner (Figure S4A–C), providing further evidence of platelet protection in vivo.

These results confirm that the galacto‐conjugation of Navitoclax to produce the Nav‐Gal prodrug serves as an effective strategy to decrease Navitoclax‐driven thrombocytopenia at physiologically relevant concentrations capable of halting cancer growth. Altogether, we conclude that galacto‐conjugated Navitoclax is effective in clearing senescent cells in vivo and can reduce its associated toxicities.

## 3 DISCUSSION

In the last decade, the use of novel genetically engineered mouse models has demonstrated that the selective elimination of senescent cells attenuates a number of age‐related pathologies and promotes the healthspan and lifespan of mice (Baker et al., 2016). These observations resulted in the development of senotherapies—therapies aimed at interfering with cellular senescence, either by killing senescent cells (senolytics) or by inhibiting the SASP (senomorphics or senostatics) (Paez‐Ribes et al., 2019). However, specifically targeting senescent cells still remains a considerable challenge in most cases, and senotherapies are not exempt from both on‐ and off‐target toxicities, due to the activity of these agents in normal cells. Here, we report a versatile methodology for the generation of prodrugs that permits a more selective senolytic activity. We show that galacto‐conjugation of the BCL‐2 family inhibitor Navitoclax allows therapeutically relevant activity in subcutaneous tumour xenografts and orthotopic mouse models of chemotherapy‐induced senescence in the context of lung carcinoma. In addition, this strategy reduces Navitoclax‐associated thrombocytopenia in treated mice, and platelet apoptosis in human and murine blood samples.

Targeting cellular senescence by galacto‐conjugation relies on the high levels of lysosomal β‐galactosidase (SA‐β‐gal) activity present in multiple types of senescent cells. Although SA‐β‐gal is an imperfect marker of senescence, diseased tissues are often positive for this marker, particularly when senescent cells accumulate and persist in damaged areas (Hernandez‐Segura et al., 2018; Sharpless & Sherr, 2015). To exploit the accumulated pathological SA‐β‐gal activity, we previously used beads with a mesoporous silica core that were functionally capped with a homogeneous coating of galacto‐oligosaccharides, thereby allowing the encapsulation of cytotoxic drugs, including doxorubicin and Navitoclax, as well as fluorescent tracers for the detection of senescent cells. This nanotechnology‐enabled preferential cargo release within senescent cells, and the efficacy of this approach was validated in models of bleomycin‐induced pulmonary fibrosis and palbociclib‐treated melanoma and NSCLC tumours (Muñoz‐Espín et al., 2018). However, despite the emergent interest in the development of nanomedicine and the considerable technical success of an increasing number of nanotechnologies in preclinical models, only a small number of formulations have reached clinical translation. The current limitations stem from an incomplete understanding of nano‐bio interactions, potential toxicities, and challenges regarding biodistribution, such as systemic trafficking, presence of physiological barriers, mechanisms of cellular uptake, pharmacokinetics/pharmacodynamics (PK/PD), and routes and timelines of elimination (Shi, Kantoff, Wooster, & Farokhzad, 2017). In order to simplify the delivery of drugs to the lysosome of senescent cells, we recently created an OFF‐ON two‐photon fluorescent probe conjugated with an acetylated galactose for tracking cellular senescence in vivo (Lozano‐Torres et al., 2017). Acetylation is a widely used technique to markedly increase cellular permeabilization and uptake of N‐acetylated amino sugars (Lee et al., 2019).

We show here that conjugation of Navitoclax with a β‐galactosidase activity‐driven, cleavable acetylated galactose results in an effective prodrug that enhances its selective senolytic activity (over the parent drug) in a variety of cell types and senescence triggered by different stimuli, suggesting a potentially universal approach for senescent cell targeting. This includes cisplatin‐induced senescence in human (A549) and mouse (KP) lung cancer lines; human melanoma (SK‐Mel‐103) and mouse mammary gland (4T1) cancer cell lines responsive to palbociclib; doxorubicin‐induced senescent colorectal carcinoma cells (HCT116); mouse lung fibroblasts (MLg cells) subjected to X‐ray radiation and human lung fibroblasts undergoing oncogene‐induced senescence (ER:Mek IMR90 cells). Since Navitoclax is a potent small‐molecule inhibitor of BCL‐2, BCL‐X_L_, and BCL‐_W_ proteins that exerts its killing activity by inducing apoptosis (Tse et al., 2008), we also tested whether this underlying mechanism of cell death is conserved for Nav‐Gal. Similarly to Navitoclax, we observed that Nav‐Gal induces the appearance of annexin V‐positive cells in two models of chemotherapy‐induced senescence, namely cisplatin‐induced senescent A549 lung cancer cells and palbociclib‐induced senescent SK‐Mel‐103 melanoma cells, thereby indicating that senolysis by Nav‐Gal is driven by the implementation of the apoptotic programme.

After validating the use of Nav‐Gal with senescent cancer cells in culture, we focused on two in vivo models of lung carcinogenesis subjected to senescence‐inducing chemotherapy. We showed that simultaneous administration of Nav‐Gal with the *standard‐of‐care* senescence‐inducing cisplatin (widely used in the management of NSCLC patients) resulted in reduced tumour burden in SCID mice bearing subcutaneous tumour xenografts comprised of human A549 lung cancer cells. Concomitant treatment with cisplatin and Nav‐Gal also reduced tumour burden in an orthotopic transplantation model using murine KP lung adenocarcinoma cells. Nav‐Gal administration showed a strong effect in reducing tumour growth during both sequential and concomitant treatment with cisplatin. Combination treatment suggests an additive/synergistic effect between cisplatin and Nav‐Gal, as observed in our clonogenic assays and further confirmed by the calculated coefficient of drug interaction (CDI). It is important to note that neither Navitoclax nor Nav‐Gal had any relevant impact on tumour burden in the absence of cisplatin in our murine models, strongly suggesting a therapeutic activity restricted to the induction of senescence or whenever tumours contain senescent areas, but not in fully proliferative tumours. These observations reinforce the concept of Nav‐Gal as a senolytic prodrug and, more generally, open up the possibility of using senotherapies as promising adjuncts to treat cancer in combination with senescence‐inducing chemotherapies. The eradication of senescent cancer cells by Nav‐Gal may also prevent tumour progression by mitigating the senescent secretome (SASP) and its broad range of potential protumorigenic effects (Faget et al., 2019). In this regard, cancer therapies can induce senescence not only in cancer cells but also in stromal cells, and this is also the case in lung cancer patients subjected to platinum‐ and taxane‐based chemotherapy regimens (Roberson et al., 2005). Stromal senescence appears to establish an immunosuppressive microenvironment that influences and promotes tumorigenesis (Ruhland et al., 2016) and can also be a fundamental driver of the metastatic niche (Luo et al., 2016). Future studies will examine the impact of Nav‐Gal on stromal senescence. Taken together, our data reinforce the concept that combination treatments comprised of senescence‐inducing radio/chemotherapy and senolytics might result in potent approaches for precision cancer management in the near future.

As with many targeted agents, Navitoclax presents clinically important and dose‐limiting haematological toxicities in clinical trials, including thrombocytopenia (Cang et al., 2015), presenting particular challenges when given in combination with cytotoxic chemotherapies which also have dose‐limiting haematological toxicities. For Navitoclax, this occurs because of the importance of BCL‐X_L_ in platelet survival, and the gradual reduction of its levels by Navitoclax promotes a change in the molecular clock that determines platelet lifespan leading to the pro‐apoptotic activity of BAK (Mason et al., 2007). In addition to thrombocytopenia, the first trial to evaluate Navitoclax in lymphoid malignancies resulted in grade III transaminitis and gastrointestinal bleed in a proportion of patients subjected to a continuous dosing schedule (Wilson et al., 2010). The modification of Navitoclax with an acetylated galactose may prevent the exposure of healthy (nonsenescent) cells to the inhibitory activity of BCL‐2 family protein members and, thereby, reduce unwanted side effects. In support of this, our IC_50_ experiments with A549 cells treated with cisplatin indicated that the enhanced senolytic index of Nav‐Gal when compared to Navitoclax is mainly due to reduced cytotoxicity in the absence of senescence induction, suggesting that the prodrug is not efficiently activated in nonsenescent A549 cells. Also, we noted that although the percentage of annexin V‐positive senescent cancer cells at a high concentration of both Navitoclax and Nav‐Gal is similar and results in the complete eradication of these cells, nonsenescent cancer cells were substantially more protected from the induction of apoptosis when exposed to Nav‐Gal, indicating enhanced selective sensitivity for Nav‐Gal for senescent cells. Importantly, we have observed that Nav‐Gal results in less thrombocytopenia (compared with Navitoclax) in daily‐treated mice. We also show that Nav‐Gal reduces apoptosis in platelets, using both human and mouse blood samples. A recent study has shown that another Navitoclax derivative, namely DT2216, exerts anti‐tumour activity by targeting BCL‐ X_L_ to the VHL E3 ligase for proteolytic degradation (Khan et al., 2019). However, distinctly to Nav‐Gal, DT2216 has not been designed for targeting senescent cells, but for reducing thrombocytopenia (as platelets are characterized by poor VHL E3 ligase expression) while maintaining anti‐cancer properties. In addition, DT2216 specifically targets BCL‐X_L_, but not BCL‐2 or BCL‐W, which may also hinder its potential senolytic activity.

In summary, we have synthesized a potent senolytic prodrug that can be used to kill multiple types of senescent cells. We propose galacto‐conjugation as a versatile strategy that might be expanded to other cytotoxic agents and senolytic drugs to develop more specific, next‐generation senolytics. As a *proof‐of‐principle*, we show that galacto‐conjugated Navitoclax (Nav‐Gal) selectively kills a variety of human and murine cell types undergoing senescence by different stressors in vitro and that it has therapeutic efficacy in combination with cisplatin in murine NSCLC models. Importantly, we also demonstrate that galacto‐conjugation of senolytics can reduce potential toxicities of the conjugated drug. The development of galacto‐conjugated prodrugs has considerable promise for improving cancer treatment, as well as other human senescence‐related disorders.

## 4 EXPERIMENTAL PROCEDURES

### 4.1 Synthesis of Nav‐Gal prodrug

For the synthesis of Nav‐Gal prodrug, 40 mg (0.04 mmol) of Navitoclax (Eurodiagnostico), 25 mg (0.06 mmol) of 2,3,4,6‐tetra‐O‐acetyl‐α‐D‐galactopyranosyl‐bromide (Sigma) and 10.5 mg (0.07 mmol) of K_2_CO_3_ (Sigma) were mixed. Anhydrous acetonitrile (Sigma) was added, and the mixture was stirred at 70°C for 3 hr under argon‐enriched atmosphere. The solvent was eliminated under vacuum. The product was purified by flash chromatography on silica gel (Sigma), from hexane‐ethylacetate (3:7 v/v; Scharlab) to hexane‐ethylacetate (7:3 v/v) used as eluent. Purified Nav‐Gal was obtained as a yellow powder in 35% yield. For large stocks used in mouse experiments, the reactions were performed in different batches. Once the desired amount was obtained, all batches were pooled and purified on a single flash chromatography column as described above.

### 4.2 Characterisation of Nav‐Gal prodrug

Nav‐Gal was characterized by conventional techniques. For samples preparation, pure Nav‐Gal was dissolved in deuterochloroform (CDCl_3_; Sigma) and transferred to a Wilma NMR tube (Sigma). Samples were immediately analyzed by proton nuclear magnetic resonance (^1^H NMR), Carbon‐13 nuclear magnetic resonance (^13^C NMR) and homonuclear bidimensional correlated spectroscopy (COSY NMR). NMR spectra were acquired with a Bruker FT‐NMR Avance 400 (Ettlingen, Germany) at 300 K and analyzed with MestReNova 6.0 software. Chemical shifts are expressed as δH (ppm) relative tetramethylsilane (TMS). Attenuated total reflectance (ATR) was performed in a Bruker Tensor 27 FT‐IR (Ettlingen, Germany) at room temperature and analyzed with OPUS Data Collection Program (V 1.1). Finally, high resolution mass spectrometry (HRMS) was recorded with an ABSciex TRIPLETOF T5600.

### 4.3 Hydrolysis reaction of Nav‐Gal studies

For hydrolysis reaction studies, aqueous solutions of Navitoclax and Nav‐Gal at a concentration of 10^–5^ M (pH 7) in 0.01% DMSO were prepared. Human β‐galactosidase (Biotechne, R&D Systems) was then added to Nav‐Gal solutions to a final concentration of 8 ng/µl, and chromatograms were acquired after complete reaction (*λ*_exc_ = 365 nm) with a Waters 1525 binary HPLC pump equipped with a Waters 2990 diode array detector. Chromatograms were obtained using Empower 3 software. Conditions: KromasilC18 column, 0.9 ml/min, (H_2_O (0.1% acetic acid)): MeOH gradient elution: 50:50 3 min, 40:60 7 min, 30:70 10 min, 20:80 5 min, 10:90 3 min, 0:100 3 min. Data analysis was performed using OriginPro8 software.

### 4.4 Cells and reagents

The human lung carcinoma cell line A549 was obtained from the European Collection of Authenticated Cell Cultures (ECACC). SK‐MEL‐103 (human melanoma), 4T1 (breast cancer), HCT116 (human colorectal carcinoma) and MLg (mouse lung fibroblastic) cell lines were obtained from the American Type Culture Collection (ATCC). The murine KP lung adenocarcinoma cell line L1475 was derived from *KrasLSL‐G12D/+;p53Fx/Fx* mice (Jackson et al., 2005) and transduced with MSCV‐luciferase‐hygromycin retrovirus, as previously described (Turrell et al., 2017). A549, SK‐MEL‐103, 4T1 and L1475(luc) cell lines were maintained in DMEM, and supplemented with 10% FBS (Sigma). HCT116 cells were grown in McCoy's 5A medium (Thermo Fisher Scientific) supplemented with 10% FBS. MLg cells were cultured in DMEM‐F‐12 culture media (Sigma) supplemented with 2 mM l‐Glutamine and 10% FBS. ER:Mek IMR90 cells were cultured in phenol red‐free DMEM (Sigma) supplemented with 10% FBS, 2 mM l‐Glutamine and 1 mM sodium pyruvate (Sigma). All cells were incubated in 20% O_2_ and 5% CO_2_ at 37°C. Cells were routinely tested for mycoplasma using the universal Mycoplasma Detection Kit  (ATCC) or by RNA‐capture ELISA.

For senescence induction, A549 cells were treated with 15 µM of cisplatin (Stratech) for 10 days. SK‐MEL‐103 and 4T1 cells were treated with 5 µM palbociclib (Eurodiagnostico) for 7 days. HCT116 cells were treated with 100 nM doxorubicin (Carbosynth) for 72 hr. MLg cells were irradiated with 10 Gy and used for viability assays 10 days later. ER:Mek IMR90 cells were treated with 200 nM 4‐hydroxytamoxifen for 72 hr and used for viability assays 2 days later.

For experiments with cells, cisplatin (Stratech) was reconstituted in sterile PBS; palbociclib (Eurodiagnostico), Navitoclax (Stratech) and Nav‐Gal were reconstituted in DMSO. For in vivo experiments, cisplatin was reconstituted in saline; Navitoclax was formulated in 10% ethanol, 30% polyethylene glycol 400 and 60% Phosal PG, and Nav‐Gal was reconstituted in DMSO and further diluted in saline.

### 4.5 GLB1 downregulation

For transient downregulation of GLB1, a total of 30,000 control or 50,000 senescent A549 and SK‐MEL‐103 cells were plated per well in a 24‐well plate. The next day, cells were transfected with TriFECTa^®^ Kit DsiRNA Duplex siRNAs (Integrated DNA Technologies) hs.Ri.GLB1.13.1 (siRNA1), hs.Ri.GLB1.13.3 (siRNA2) or scrambled siRNA, using Lipotectamine RNAiMAX Reagent (Thermo Fisher Scientific) as per manufacturer's instructions. After 48 hr, RNA was extracted using the RNeasy Mini Kit (Qiagen) and cDNA was synthesized with the High‐Capacity RNA‐to‐cDNA™ Kit (Thermo Fisher Scientific).

Gene expression of GLB1 and ACTB genes was measured by quantitative real‐time PCR performed on a QuantStudio thermocycler (Applied Biosystems) following Luna^®^ Universal qPCR Master Mix (New England Biolabs) protocol and amplification parameters, and using predesigned KiCqStart^®^ SYBR^®^ Green Primers H_GLB1_1 and H_ACTB_1 pairs (Sigma‐Aldrich). Relative quantification was carried out using 2−ΔΔCt methodology.

### 4.6 Cell viability and apoptosis analysis

For A549, SK‐MEL‐103, 4T1, HCT116 and ER:Mek IMR90 cell lines, control and senescent cells were seeded in flat‐bottom‐clear 96‐well plates at a density of 6,000–8,000 and 4,000–6,000 cells per well, respectively. The following day cells were treated with serial dilutions of Nav‐Gal or Navitoclax in 0.2% FBS‐containing media. Viability was assessed 48 or 72 hr later after two hours of incubation at 37°C with CellTiter‐Glo^®^ Luminescent Cell Viability Assay (Promega) or CellTiter‐Blue^®^ Cell Viability Reagent (Promega). Raw data were obtained by measuring luminescence in a VICTOR Multilabel Plate Reader (Pelkin Elmer) or fluorescence at an excitation/emission wavelength of 560 nm/590 nm in an Infinite 200 PRO Multimode Spectrophotometer (TECAN).

For MLg cells, control and senescent cells were seeded in a 12‐well plate at a density of 80,000 and 60,000 cells per well respectively, and cells were treated with three to five different increasing concentrations of Nav‐Gal or Navitoclax on the following day. After 72 hr, viability was assessed with CellTiter‐Blue^®^ Cell Viability Reagent (Promega) as described above.

To determine the induction of apoptosis after the treatment with Navitoclax and Nav‐Gal, 4,000 senescent and 6,000 nonsenescent A549 or SK‐Mel‐103 cells were seeded in 96‐well plates. 24 hr later, annexin V Fluorescent Reagent (Essen Bioscience) was added to the media, and after a first scan, the cells were treated with vehicle (DMSO), 11 µM Navitoclax or 11 µM Nav‐Gal for 48 hr. Images were collected every 2 hr with an Incucyte^®^ S3 Live‐Cell Analysis System microscope (Essen Bioscience) over time. A total of 2 pictures per well were analyzed using the IncuCyte ZOOM™ software analyser, and total and annexin V‐positive cells were counted using ImageJ software.

### 4.7 Clonogenic assay

For clonogenic potential analysis, 10,000 A549 cells were seeded per well in a 6‐well plate and allowed to attach overnight. In a first approach (concomitant treatment), cells were then treated with increasing and concomitant concentrations of cisplatin and Navitoclax or Nav‐Gal. In a second approach (sequential treatment), cells were first treated with increasing concentrations of cisplatin for 7 days, and then Navitoclax or Nav‐Gal were added for 7 additional days. Fresh drug was added every 2–3 days, and 10 days later, colonies were washed with PBS and fixed with 4% PFA for 10 min. They were then permeabilized using ice‐cold methanol for 20 min, and left to dry. Colonies were then stained with 0.5% crystal violet for 30 min, and excess was removed by thoroughly washing three times with deionised water. Colonies were imaged using a BioRad GelDoc XR+ machine (BioRad Technologies). To calculate mean clonogenic potential, staining of colonies was subsequently diluted in 10% acetic acid and optical density (OD) was measured at 595 nm using an Infinite 200 PRO Multimode Spectrophotometer (PECAN) microplate reader. The blank (10% acetic acid) was subtracted from each OD measurement, and the average value for each experimental condition was calculated and normalized against clonogenic potential value obtained in no‐treatment condition.

To analyze the synergistic effect of the concomitant treatment of cisplatin and the senolytics Navitoclax and Nav‐Gal, we calculated the co‐coefficient of drug interaction (CDI) using the normalized clonogenic potential values of the individual and concomitant treatments at different concentrations. CDIs were calculated as ratios following the formula CDI = (AB)/(AxB), where AB is the clonogenic potential value of the concomitant treatment with the two drugs combined at a specific concentration, and A and B are the clonogenic potential values of the individual treatments of each drug at such concentration. Following this formula, a CDI > 1 indicates that the drugs are antagonistic, a CDI = 1 indicates that the drugs are additive and a CDI < 1 indicates that the drugs are synergistic, with values closer to 0 indicating higher synergy between the drugs. This effect can depend on the ratio and molarity of drugs used, and for this reason, CDIs were calculated and plotted at different molar ratios of cisplatin and Navitoclax or Nav‐Gal.

### 4.8 Immunoblotting

Cell lysis was performed using RIPA buffer (Sigma) supplemented with phosphatase inhibitors (PhosSTOP™ EASYpak Phosphatase Inhibitors Cocktail, Roche) and protease inhibitors (cOmplete™ Protease Inhibitor Cocktail, Roche). Proteins were quantified and separated by SDS‐PAGE and transferred to polyvinylidene difluoride (PVDF) membranes (Millipore) according to standard protocols. Membranes were immunoblotted with antibodies against p21 and p53 from Santa Cruz Biotechnology, and phospho‐Rb (pRBS780) from Cell Signaling. After incubation with the primary antibody overnight, membranes were washed and incubated with secondary HRP‐conjugated AffiniPure antibodies (Jackson ImmunoResearch) for 1 hr at room temperature and subsequently incubated with Enhanced Chemiluminescence Detection solution (Amersham). Membranes were placed on X‐ray films and processed using a Xograph Compact X4 automatic processor.

### 4.9 Mouse experiments

All mice were treated in strict accordance with the local ethical committee (University of Cambridge Licence Review Committee) and the UK Home Office guidelines. For the orthotopic model experiment, 5–6 weeks old C57BL/6J mice were transplanted in the lungs with 3 × 10^5^ syngeneic luciferase‐expressing KP (L1475(luc)) lung tumour cells via tail‐vein injection. All mice were maintained in ventilated cages within a specific pathogen free animal facility. Luminescence values were recorded 5 days later after i.p. injection with D‐luciferin (150 mg/kg body weight, PerkinElmer) using an IVIS Spectrum Zenogen machine (Caliper Life Sciences) and analyzed with Living Image software, and then randomized into the different experimental groups. Mice were treated with either vehicle, 1 mg/kg CDDP (via i.p. injection) every 3 days and 85 mg/kg Nav‐Gal (via i.p. injection) for two consecutive days after each CDDP administration. Luminescence values were recorded again as described above on day 5 and 15, when treatment was finished, all mice were culled by anaesthetic overdose, and lungs were collected for subsequent histological analyses.

To establish subcutaneous tumour xenografts, 5–7 weeks old SCID (CB17‐Prkdc^scid^/J) females were injected subcutaneously with 4 × 10^6^ A549 cells in each flank. Tumours were measured with callipers every 2–3 days, and the tumour volume was calculated with the formula *length* × *width^2^*/*2*. When tumour volume reached an average of 100 mm^3^, mice were randomized and assigned to one of the control or therapy groups. Therapy was initiated with either vehicle, 1.5 mg/kg cisplatin (CDDP, via intraperitoneal (i.p.) injection) three times per week, 100 mg/kg Navitoclax (via oral gavage) 5 days ON/2 days OFF, 85 mg/kg Nav‐Gal (via i.p. injection) 5 days ON/2 days OFF, or a combination of the mentioned drugs. Mice were culled by cervical dislocation after 3 weeks of treatment.

For platelet toxicity analysis, wild‐type C57BL/6J mice were treated daily with 100 mg/kg Navitoclax (via oral gavage), 85 mg/kg Navitoclax (via i.p. injection) or 85 mg/kg Nav‐Gal (via i.p. injection) for 10 consecutive days.

To analyze platelet count in mice, blood was collected by cardiac puncture during isoflurane anaesthesia in xenograft experimental mice, and by superficial vessel puncture in wild‐type C57BL/6J experimental mice. Blood was collected into anticoagulating Microvette^®^ tubes (Sarstedt), and platelet levels were immediately measured using a Scil Vet abc Plus hematology analyzer (Horiba).

### 4.10 Histology

SA‐β‐Gal staining was performed in frozen tissue sections using the Senescence β‐Galactosidase Staining kit (Cell Signaling), following the manufacturer instructions. Briefly, whole tissue was fixed at RT for 15 min with a 2% formaldehyde and 0.2% glutaraldehyde, washed and incubated for 6 hr at 37°C with the staining solution containing X‐gal in N‐N‐dimethylformamide (pH 6.0). Tissues were subsequently counterstained with nuclear fast red, dehydrated and mounted. For immunohistochemistry, 5 mm paraffin sections were deparaffinized and re‐hydrated, and slides were incubated with anti‐p21 (Abcam) and Ki67 (Abcam) antibodies at 4°C overnight, or processed for TUNEL staining using the DeadEnd™ fluorometric TUNEL System (Promega) as per manufacturer's instructions. Immunohistological reaction was of p21, and Ki67 was developed using 3,3‐diaminobenzidine tetrahydrochloride (DAB), and nuclei counterstained with haematoxylin. For immunofluorescence, frozen tissue sections were either processed for TUNEL staining using the above mentioned kit or alternatively fixed in 4% PFA for 10 min, permeabilized with 0.5% Triton™ X‐100 for 3 min and subsequently blocked with Normal Donkey Serum for 1 hr at room temperature. They were then probed with anti‐p21 (Abcam) and Ki67 (Abcam) antibodies at 4°C overnight. The following day, sections were incubated with Anti‐Rat IgG 555 and Anti‐Rabbit IgG 488 secondary antibodies (Cell Signaling Technologies), briefly incubated in DAPI‐containing PBS, and mounted using Fluoromount‐G™ Mounting Medium. Images were obtained using an AxioScan Slide Scanner (Zeiss) and processed with ZEN 2 Blue Edition software (Zeiss). Positive signal for p21, Ki67 and TUNEL was quantified with ImageJ.

### 4.11 Platelet apoptosis analysis

For human platelets apoptosis assay, blood from three healthy volunteers was extracted according the UPV ethics procedures and processed essentially as shown in Vogler et al., 2011). For mouse platelet apoptosis assay, blood from five wild‐type C57BL/6J mice was collected by cardiac puncture in accordance with the University of Cambridge ethical committee and the UK Home Office guidelines. Samples were collected in nonvacuum citrate tubes (DH medical material), and blood in control tubes was diluted in Tyrodes Buffer. Blood in treated samples was mixed with 2X drug solution (either Navitoclax or Nav‐Gal) in RPMI (10% FBS). Control and treated specimens were incubated for 5 hr in 20% O_2_ and 5% CO_2_ at 37°C. Treated samples were diluted in Tyrodes Buffer, and then all specimens were mixed with Annexin Binding Buffer (Thermo Fisher Scientific) and incubated for 10 min with Annexin V‐FITC (Immunostep) and CD‐41‐PE (Immunostep) conjugated antibodies. A23187 (Sigma), a calcium ionophore, was also added in parallel control samples to induce cell death and be used as a positive control. Samples were finally diluted in Annexin Binding Buffer (Thermo Fisher Scientific), and data were acquired using an FC500 MPL Flow Cytometer (Beckman‐Coulter) for human samples and an LSR Fortessa cell analyzer running the FACSDiva software (BD Biosciences) for mouse samples. FlowJo 10.3 software was used to analyze the results.

### 4.12 Statistical analysis

Statistical analyses were performed as described in the figure legend for each experiment. Statistical significance was determined by one‐ and two‐way ANOVA, Student's *t* tests and Bonferroni post hoc tests using Prism 5 software (GraphPad) as indicated. A *p*‐value below .05 was considered significant and indicated with asterisk: *p < .05, **p < .01 and ***p < .005.

## CONFLICT OF INTERESTS

R.M.‐M. and M.S. are founders and advisors of Senolytic Therapeutics, Inc. C.P.M is an AstraZeneca employee. The remaining authors declare no competing interests.

## AUTHOR CONTRIBUTIONS

E.G.‐G., M.P.‐R. and B.L.‐T. performed most of the experiments and contributed to the experimental designs, data analysis, discussion and writing. In particular, E.G.‐G. and M.P.‐R. performed the most of the in vivo experiments and some of the experiments with in vitro cultured cells, and B.L.‐T. developed the prodrug and performed its synthesis, characterization and hydrolysis reaction studies together with J.F.‐B., as well as the ex vivo analysis of platelet apoptosis with human blood samples and in vitro experiments with 4T1 cells. D.M. performed the ex vivo analysis of platelet apoptosis in mice and contributed to animal experiments, experimental designs, data analysis and discussion. J.R.W. carried out Incucyte Imaging System experiments, cell viability assays and contributed to data analysis. C.G.‐L. helped with histological and SA‐β‐gal analyses from tumour sections; H.O. performed cell viability assays and contributed to data analysis. S.M.‐B. assisted with initial IC50 experiments. Z.Z. helped with immunofluorescence analyses of orthotopically transplanted lungs; A. L.‐V. performed some of the experiments with in vitro cultured cells and characterization of cellular senescence in cell lines. M.R. performed IC50 experiments with SK‐Mel‐103 cells together with B.L.‐T. L.F. helped with pharmacology analyses. C.P.M. generated KP reagents and provided guidance with in vivo work. M.S. provided expertise on cellular senescence, and contributed to the discussion. G.J.D provided translational and clinical oversight and contributed to writing. R.M.‐M., A.B. and F.S. supervised the chemical synthesis of the prodrug and contributed to data analysis and discussion. D.M.‐E. designed and supervised the biological studies, analyzed the data and wrote the manuscript. All authors revised and commented on the manuscript.

## Supporting information

Figure S1

Figure S2

Figure S3

Figure S4

Figure S5

Figure S6

## Data Availability

The data that support the findings of this study are available from the corresponding author upon reasonable request.
