# Supplementary figures and images for: Galacto‐conjugation of Navitoclax as an efficient strategy to increase senolytic specificity and reduce platelet toxicity
Source: Aging Cell. 2020 Mar 31;19(4):e13142. doi: 10.1111/acel.13142 (PMC7189993; doi:10.1111/acel.13142)

**Figure S1.**

**A**

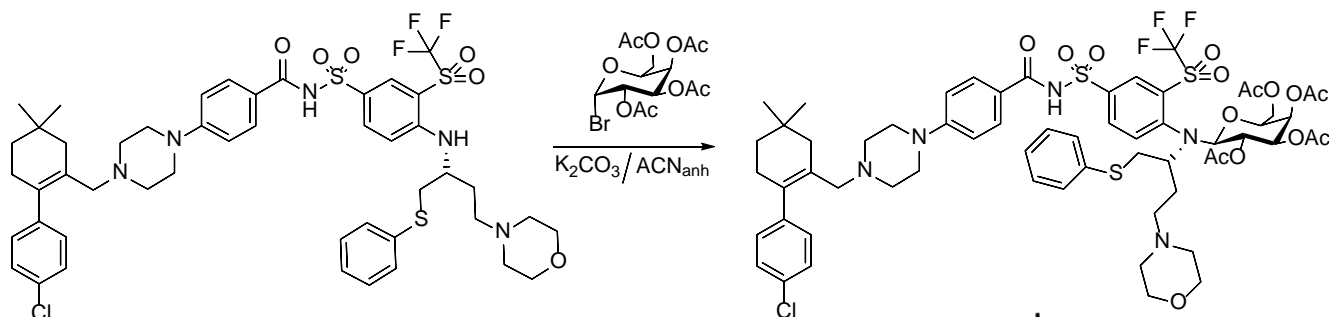

**B**

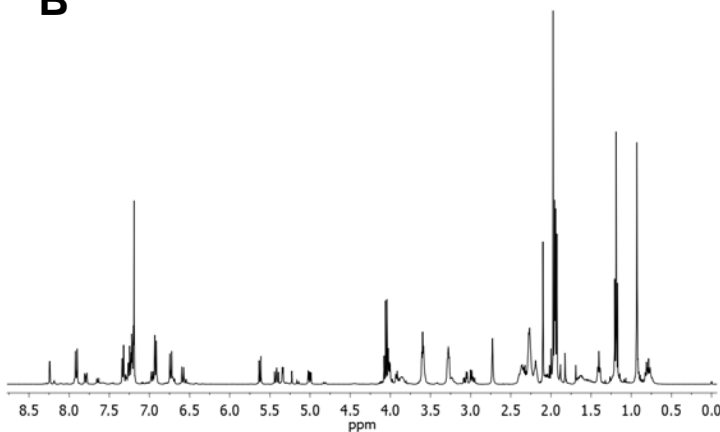

**C**

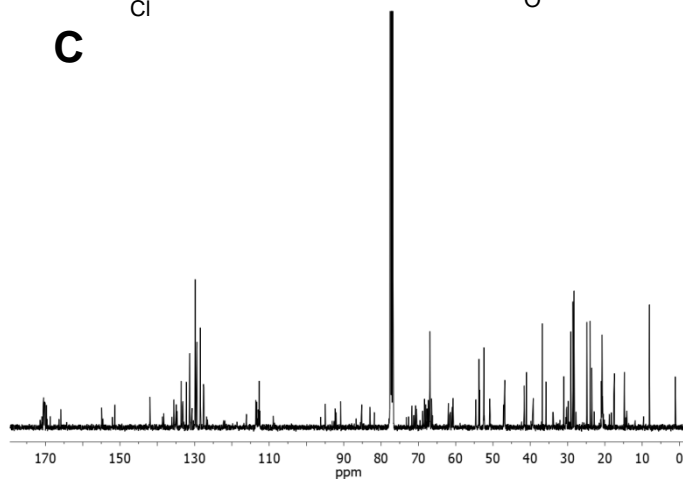

**D**

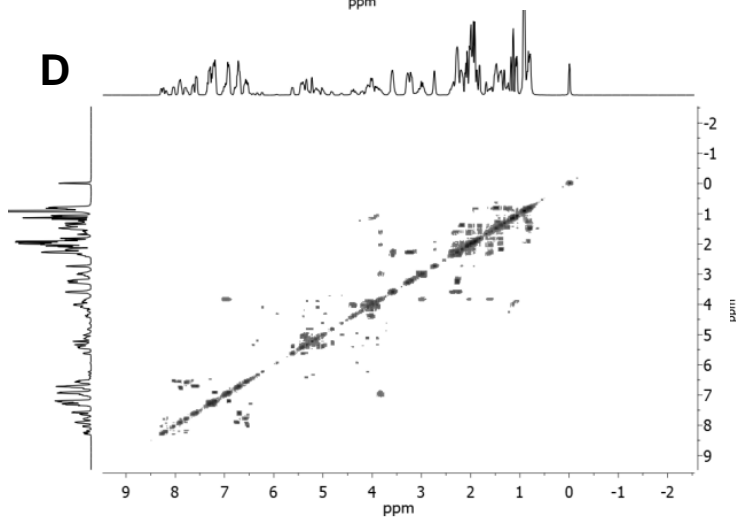

**E**

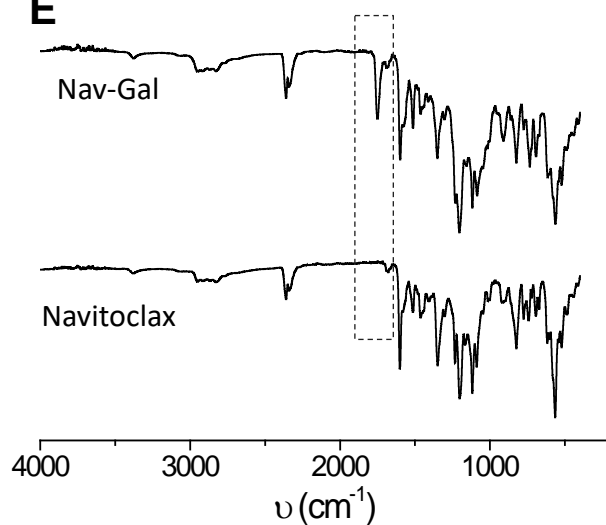

**F**

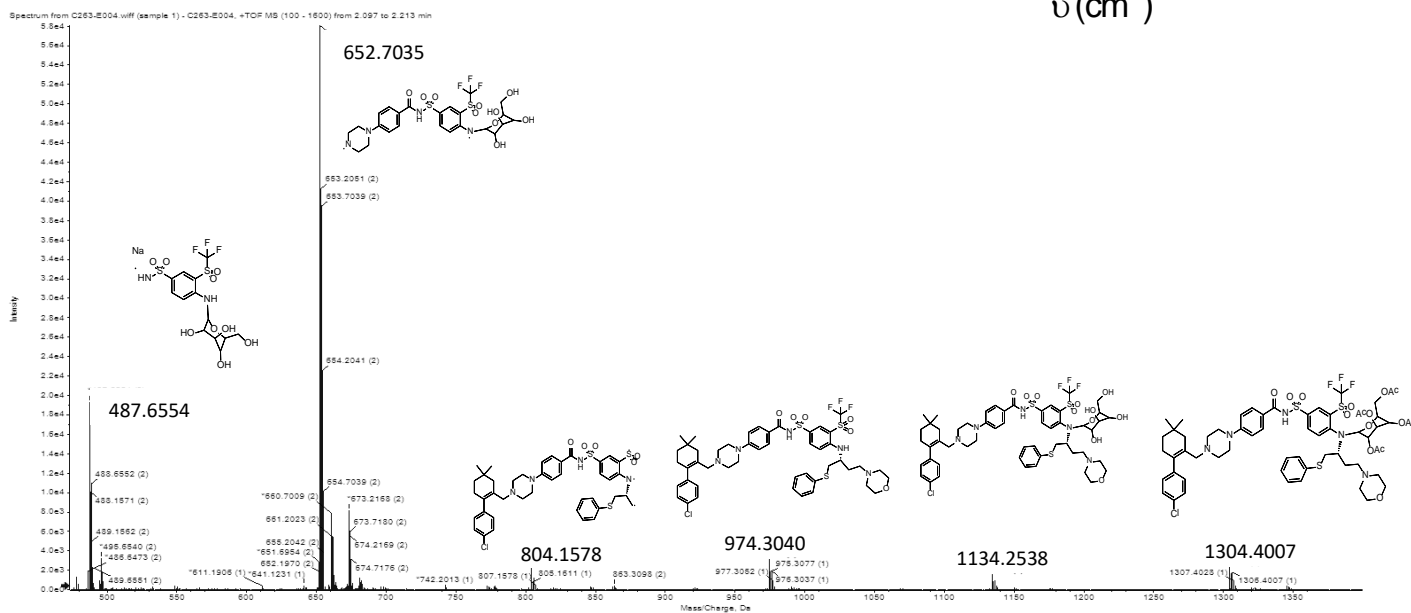

Supplement: Supplementary file 1 — Figure S1 [file ACEL-19-e13142-s001.pdf]

**Figure S2.**

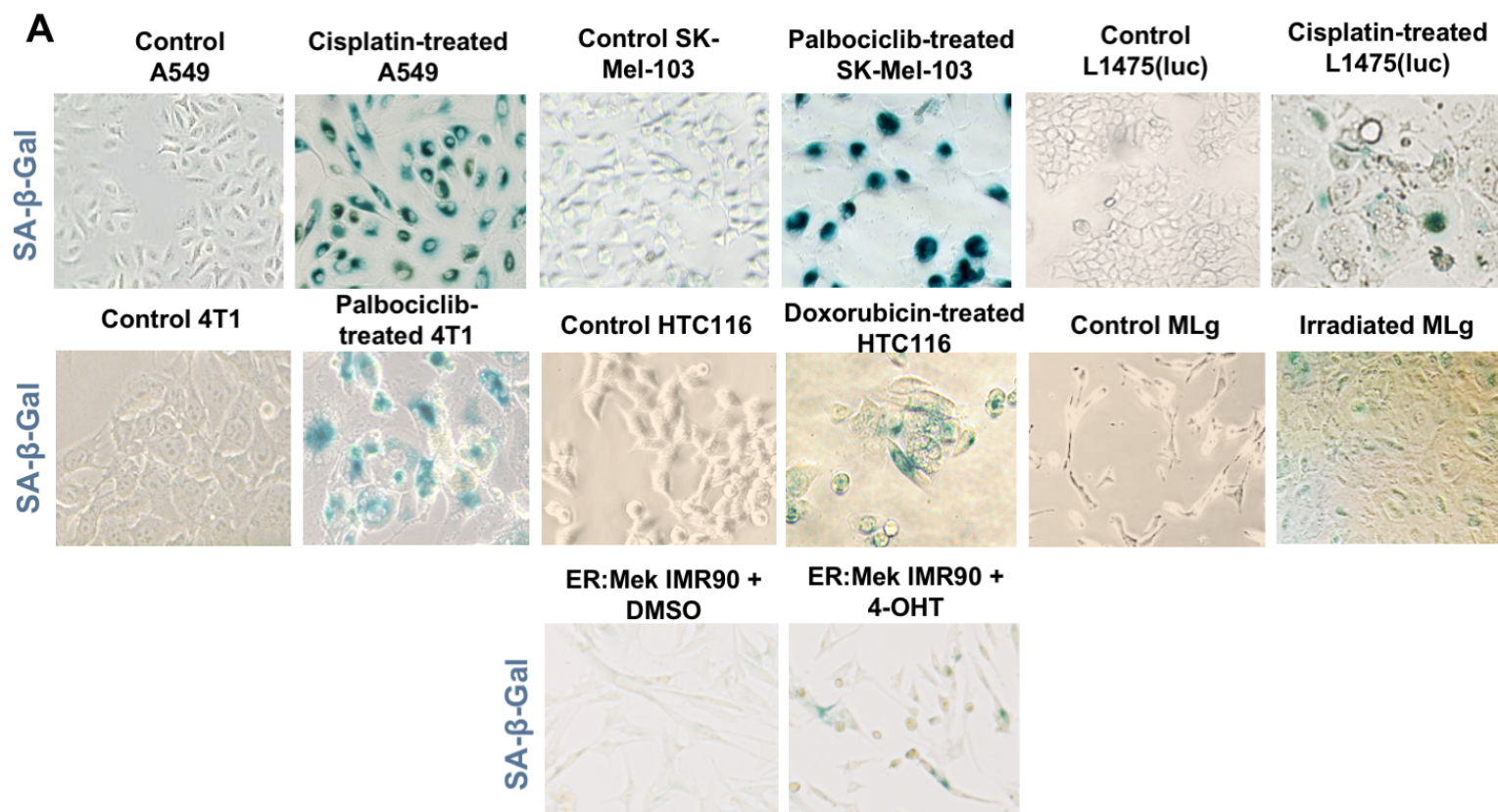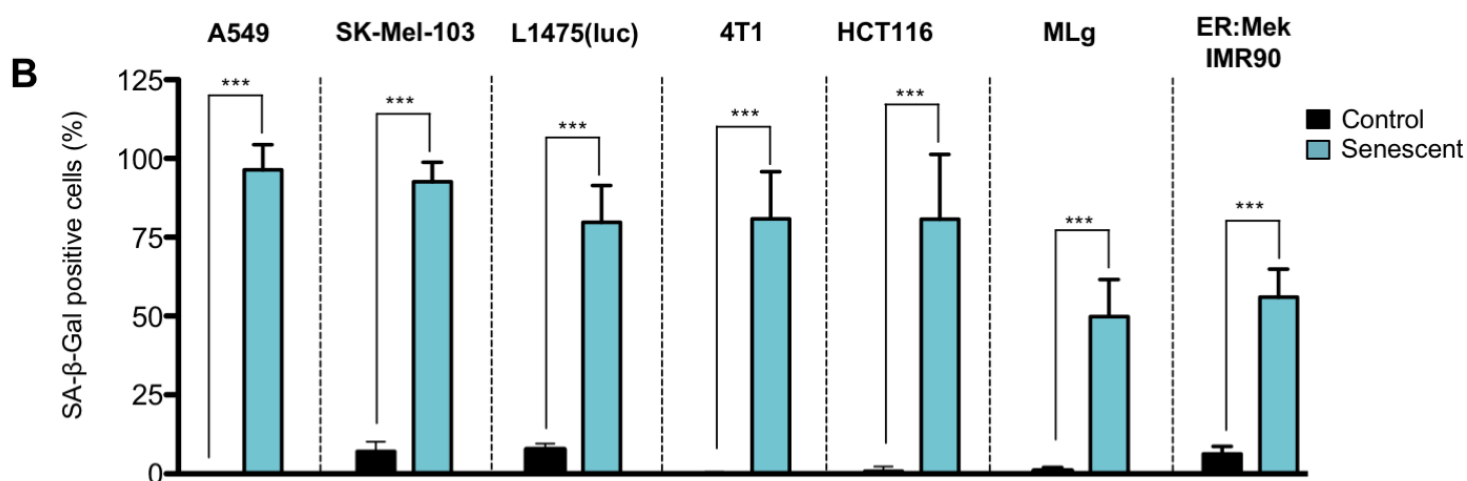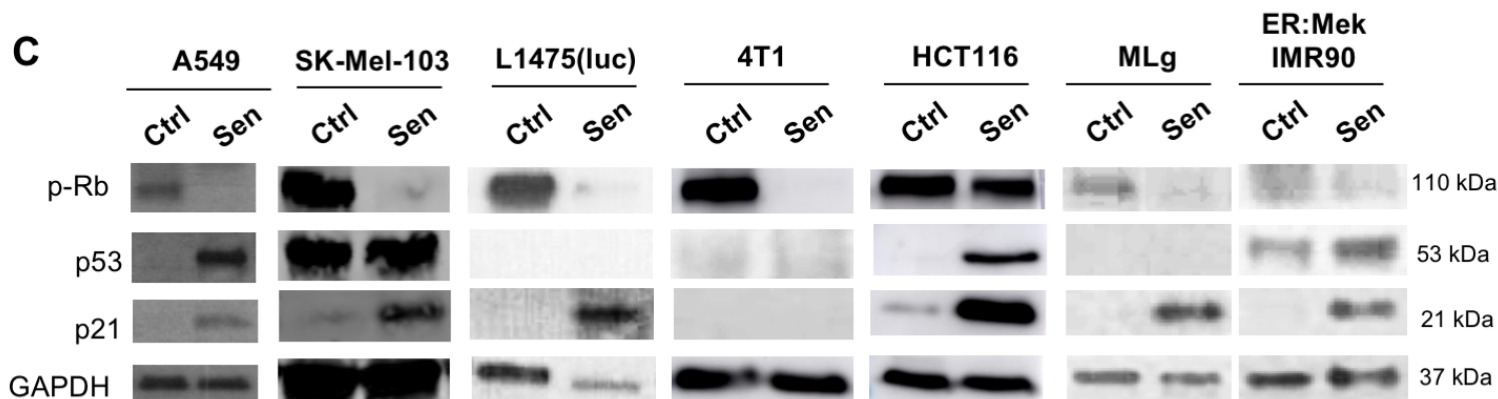

Supplement: Supplementary file 2 — Figure S2 [file ACEL-19-e13142-s002.pdf]

Figure S3.

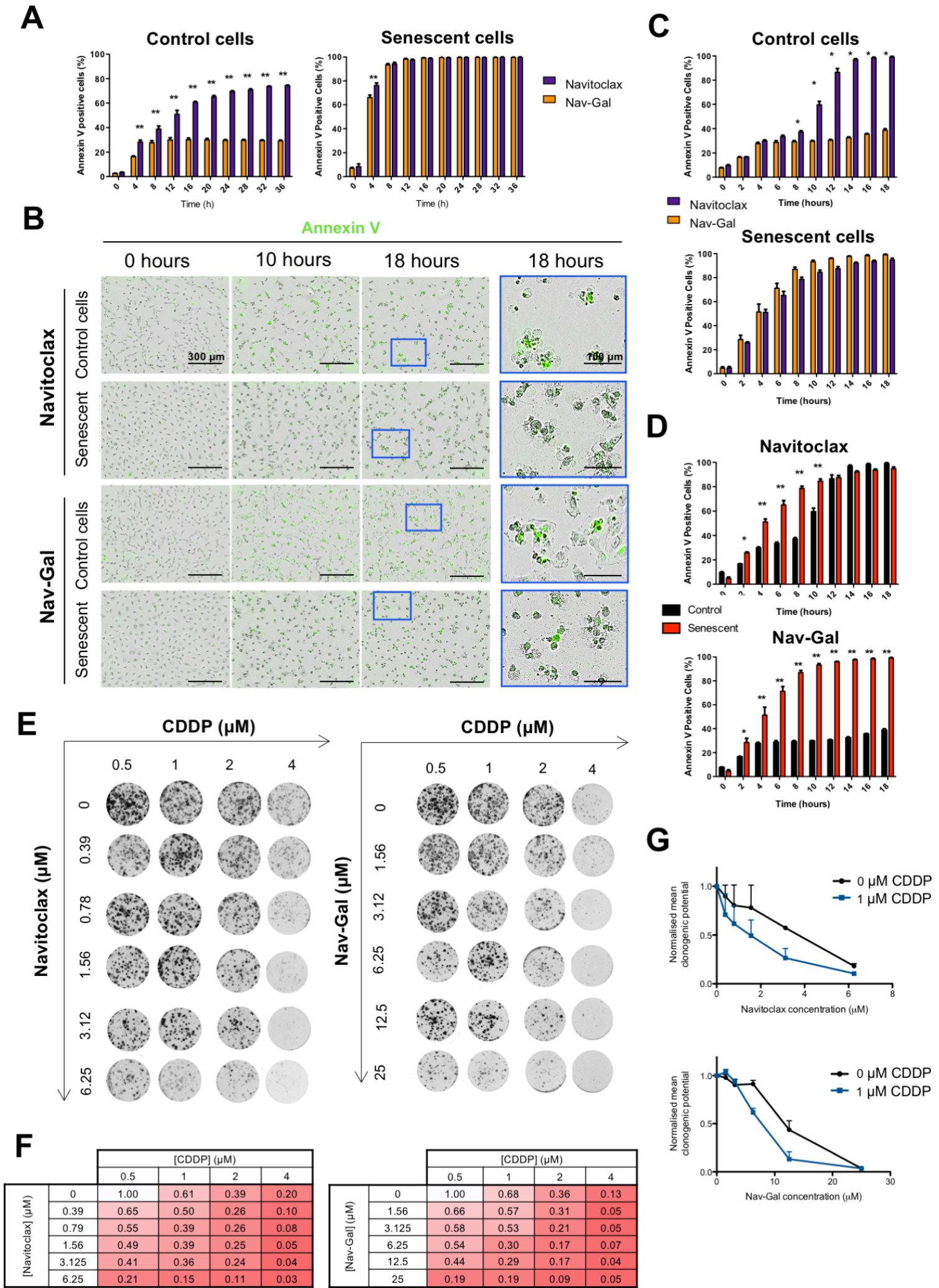

Supplement: Supplementary file 3 — Figure S3 [file ACEL-19-e13142-s003.pdf]

Figure S4.

A

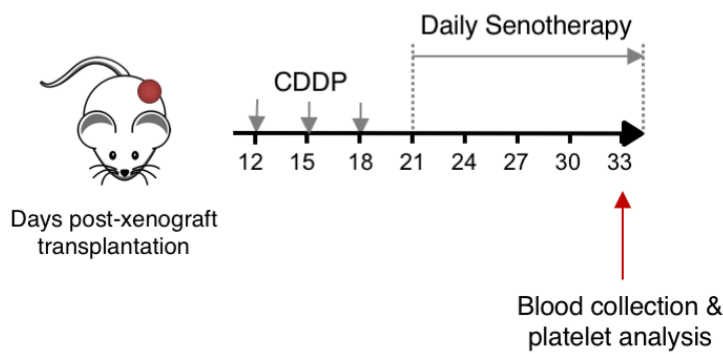

B

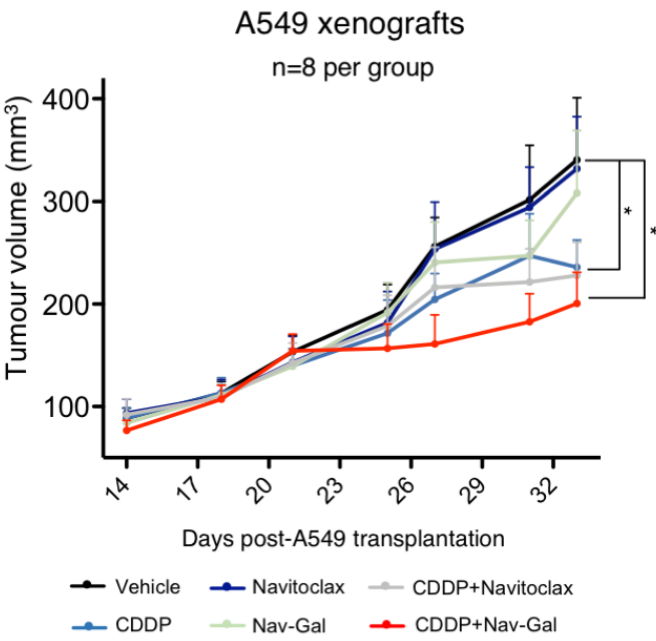

C

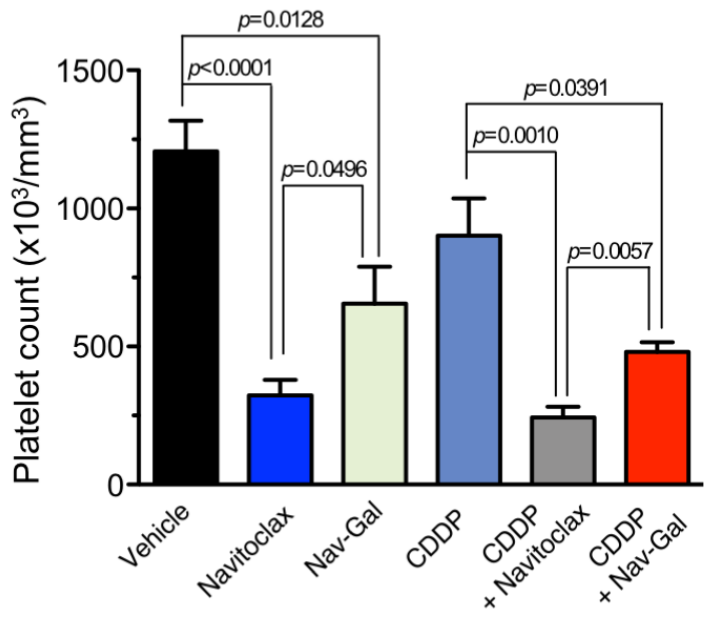

Supplement: Supplementary file 4 — Figure S4 [file ACEL-19-e13142-s004.pdf]

Figure S5.

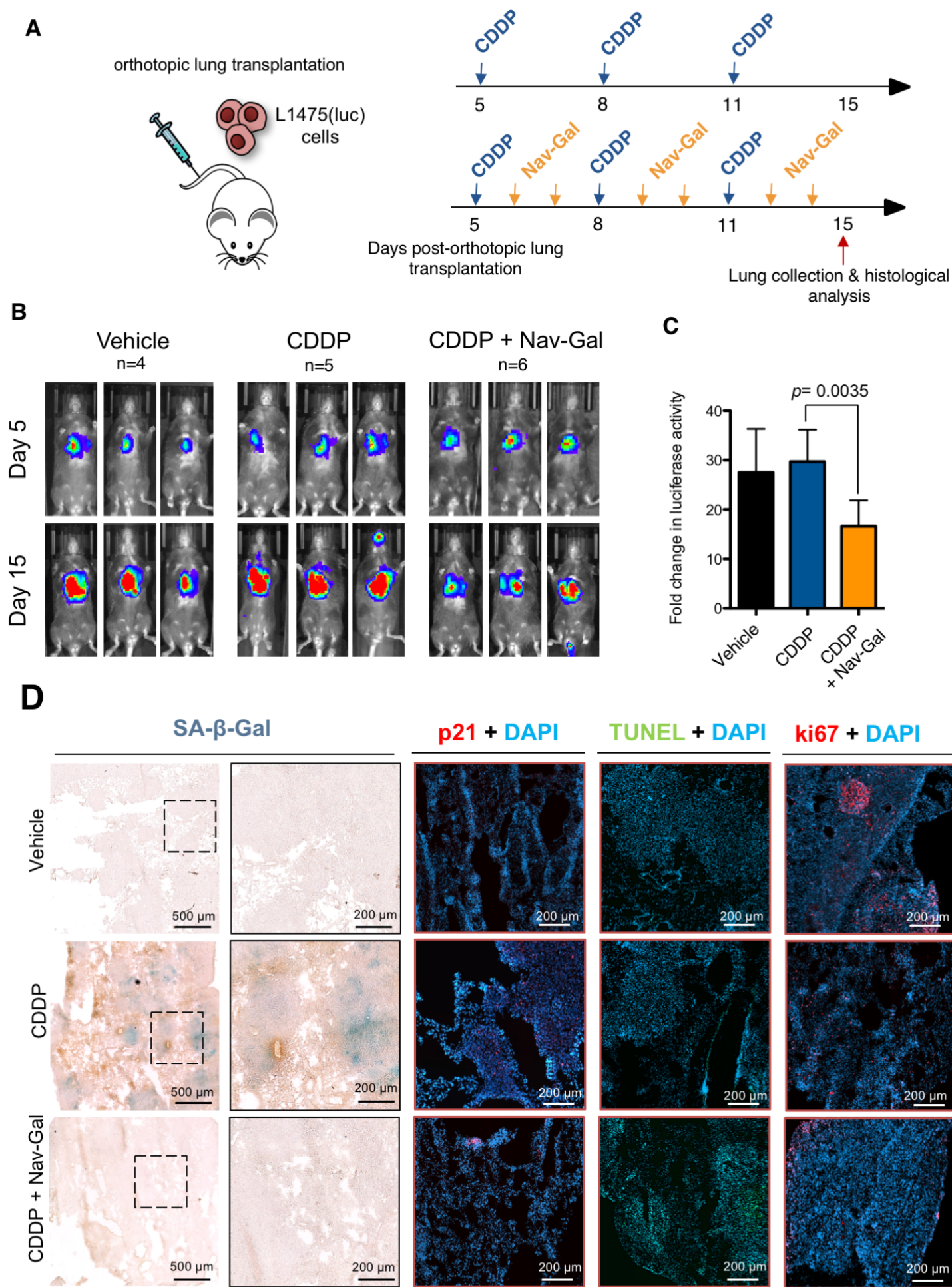

Supplement: Supplementary file 5 — Figure S5 [file ACEL-19-e13142-s005.pdf]

**Figure S6.**

**A**

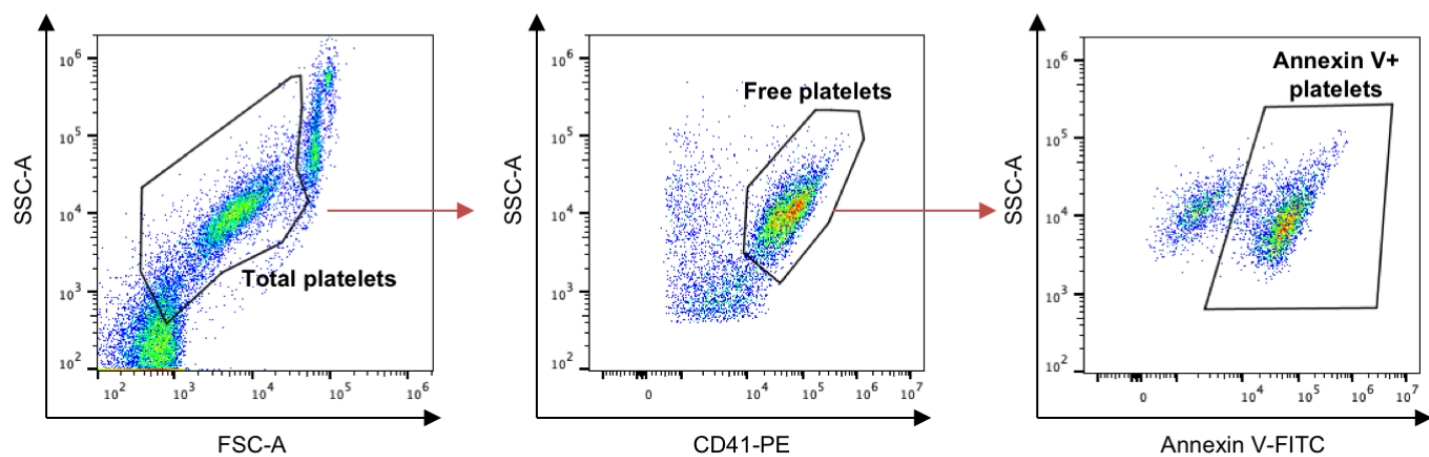

**B**

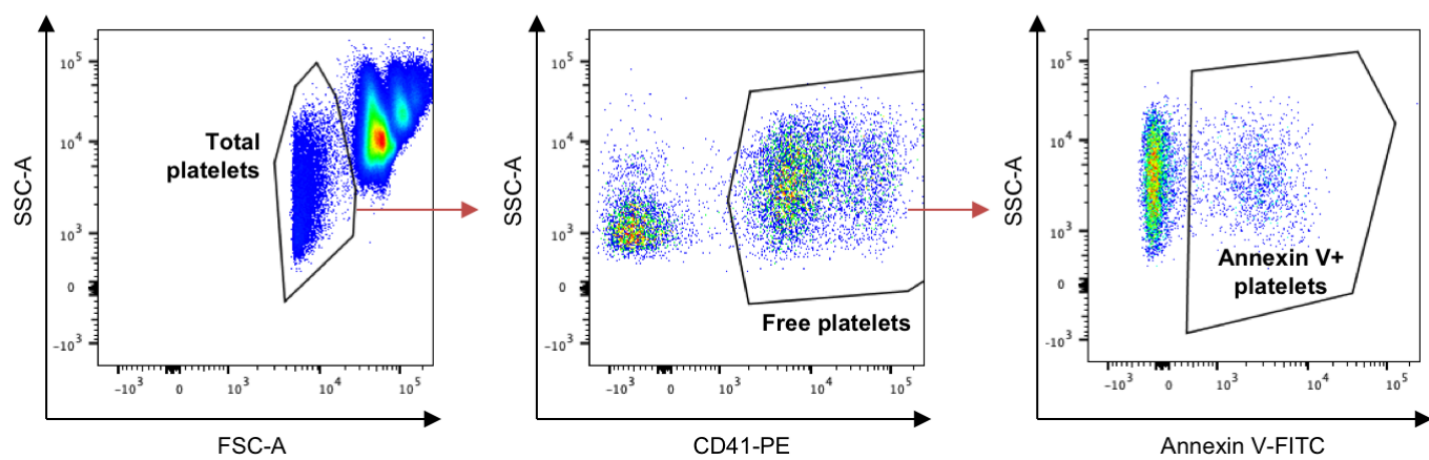

Supplement: Supplementary file 6 — Figure S6 [file ACEL-19-e13142-s006.pdf]
