# Supplementary material for: Galacto‐conjugation of Navitoclax as an efficient strategy to increase senolytic specificity and reduce platelet toxicity
Source: Aging Cell. 2020 Mar 31;19(4):e13142. doi: 10.1111/acel.13142 (PMC7189993; doi:10.1111/acel.13142)
Supplement: Supplementary file 7 [file ACEL-19-e13142-s007.pdf]

Galacto-conjugation of Navitoclax as an efficient strategy to increase senolytic specificity and reduce platelet toxicity

Estela González-Gualda[1,†], Marta Pàez-Ribes[1,†], Beatriz Lozano-Torres[2,3,4,5,†], David Macias[1], Joseph R Wilson III[1], Cristina González-López[1], Hui-Ling Ou[1], Sofía Mirón-Barroso[1], Zhenguang Zhang[1], Araceli Lérida-Viso[5], Juan F Blandez[2], Andrea Bernardos[2,3,4,6], Félix Sancenón[2,3,4,5], Miguel Rovira[7], Ljiljana Fruk[8], Carla P Martins[9], Manuel Serrano[7], Gary J Doherty[10], Ramón Martínez-Máñez[2,3,4,5,*], Daniel Muñoz-Espín[1,*].

[1] *CRUK Cambridge Centre Early Detection Programme.*
Department of Oncology, University of Cambridge. Hutchison/MRC Research Centre CB2 0XZ Cambridge, UK

[2] *Instituto Interuniversitario de Investigación de Reconocimiento Molecular y Desarrollo Tecnológico (IDM), Universitat Politècnica de València, Universitat de València, Valencia, Spain*

[3] *Unidad Mixta UPV-CIPF de Investigación en Mecanismos de Enfermedades y Nanomedicina, Universitat Politècnica de València, Centro de Investigación Príncipe Felipe Valencia, Spain*

[4] *CIBER de Bioingeniería, Biomateriales y Nanomedicina (CIBER-BBN). Madrid, Spain*

[5] *Unidad Mixta de Investigación en Nanomedicina y Sensores Universitat Politècnica de València, IIS La Fe, Valencia, Spain.*

[6] *Senolytic Therapeutics S.L., Parc Científic de Barcelona, Barcelona, Spain.*

[7] *Institute for Research in Biomedicine (IRB Barcelona), The Barcelona Institute of Science and Technology (BIST), Catalan Institution for Research and Advanced Studies (ICREA), Barcelona, Spain.*

[8] *Department of Chemical Engineering and Biotechnology University of Cambridge, Cambridge, UK*

[9] *Bioscience, Oncology, AstraZeneca, Cambridge, UK*

[10] *Department of Oncology, Cambridge University Hospitals NHS Foundation Trust*

Addenbrooke´s Hospital, Cambridge, UK,

[†] These authors contributed equally in this work

* Correspondence to: *D. M.-E.* dm742@cam.ac.uk and *R.M.-M.* rmaez@qim.upv.es

Supplementary Figure Legends

Figure S1. Molecular characterisation of pro-drug Nav-Gal. (A) Schematic chemical structural representation of synthesis of Nav-Gal prodrug. **(B-C)** Chemical shifts of the signals of proton **(B)** and carbon **(C)** atoms of Nav-Gal observed in ^{1}H and ^{13}C NMR recorded in a 400MHz NMR spectrometer using deuterated chloroform ($CDCl_3$) as solvent. ^{1}H NMR (400 MHz, $CDCl_3$) δ = 8.25 (d, J=2.19Hz, 1H), 7.98 (d, J=9.18Hz, 1H), 7.86 (dd, J= 2.19, 9.18Hz, 1H), 7.41-7.26 (m, 5H), 7.03 (d, J=8.43,2H), 7.01 (d, J=8.43Hz,2H), 6.80 (d, J=9.4Hz, 2H), 6.65 (d, J=9.4Hz, 2H), 5.71 (d, J=8.24 Hz,1H), 5.48 (dd, J=10.37, 8.24 Hz, 1H), 5.41 (d, J=2.72 Hz, 1H), 5.07 (dd, J=3.41, 10.4 Hz, 1H), 4.09-4.06 (m, 1H), 4.00-3.94 (m, 2H), 3.80 (t, J=4.51 Hz, 4H), 3.28 (t, J=5.15 Hz, 4H), 3.10-2.92 (m, 3H), 2.73 (s, 2H), 2.43-2.14 (m, 12H), 2.04 (s, 3H), 2.02 (s, 3H), 2.01 (s, 3H), 1.99 (s,3H), 1.82 (s, 2H), 1.69-1.50 (m, 2H), 1.4 (m, 3H), 0.99 (s, 6H) ppm. **(c)** ^{13}C NMR (400 MHz, $CDCl_3$) δ =170.24, 170.48, 170.26, 170.05, 169.21, 154.90, 151.31, 141.97, 135.55, 134.93, 133.63, 132.22, 131.34, 131.30, 131.29, 131.15, 129.83, 129.49, 129.44, 129.41, 129.39, 128.46, 127.62, 127.52, 113.60, 113.38, 112.67, 94.76, 90.80, 71.72, 70.76, 68.64, 68.35, 66.92, 60.68, 54.59, 53.79, 53.74, 53.56, 52.42, 50.86, 50.95, 46.82, 41.64, 41.00, 39.22, 36.77, 35.79, 31.00, 29.83, 29.11, 28.58, 28.26. **(D)** Homonuclear bidimensional correlated spectroscopy ^{1}H-^{1}H (2D) COSY NMR (400MHz, $CDCl_3$). Signals outside of the diagonal arises from the protons that are coupled together in neighbouring carbons. **(E)** Attenuated total reflectance (ATR) spectra of Nav-Gal and Navitoclax compounds, the signal centered at ca. 1795 cm^{-1} are assigned to the C=O stretching vibration present in Nav-Gal structure. **(F)** The high resolution mass spectra shows molecular fragments obtained after Nav-Gal ionization corroborating the chemical structure of Nav-Gal. HRMS-EI m/z: calculated:(M+H) = 1304.3979 m/z, measured:(M+H) = 1304.4001 m/z.

Figure S2. Assessment of the induction of cellular senescence in cell lines used for *in vitro* experiments. (A) Representative images of SA-β-gal staining of control and cisplatin-induced senescent A549 cells, palbociclib-induced senescent SK-Mel-103 cells, cisplatin-induced senescent $KRas^{G12D/WT}$;$p53^{-/-}$ lung cancer cells (L1475(luc)), palbociclib-induced 4T1 senescent cells, doxorubicin-induced senescent HTC116, irradiation-induced MLg fibroblastic cells and hydroxy-tamoxifen-treated ER:Mek IMR90 cells. **(B)** Quantification of SA-β-gal positive cells of control and senescent cells. **(C)** Western blot analysis of the expression of phospho-retinoblastoma (p-Rb), p53 and p21 in control and senescent cells.

Figure S3. The galacto-conjugated pro-drug Nav-Gal shows a lower induction of apoptosis of non-senescent melanoma SK-Mel-103 cells and lung cancer A549 cells, and significantly decreases clonogenic potential in combination with cisplatin. (A) Average percentage of Annexin V-positive

cells in control (left) or cisplatin (CDDP)-induced senescent A549 cells (right) exposed to Navitoclax (10 μM) or Nav-Gal (10 μM) treatment over time. **(B)** Representative images of cell viability depicting staining for Annexin V (green) of control or palbociclib-induced senescent SK-Mel-103 cells, exposed to Navitoclax (10 μM) or Nav-Gal (10 μM) treatment over time. Scale bar at lower magnification = 300 μm. Scale bar at higher magnification = 100 μm**.** **(C)** Average percentage of Annexin V-positive cells in control (top) or palbociclib-induced senescent SK-Mel-103 cells (bottom) exposed to Navitoclax (10 μM) or Nav-Gal (10 μM) treatment overtime. **(D)** Same as in **(C)** but directly comparing the effect in control and senescent cells of Navitoclax (top) or Nav-Gal (bottom) treatment. **(E)** Representative images of clonogenic survival of A549 cells exposed to increasing concentrations of CDDP for 7 days followed by the treatment of Navitoclax (left) or Nav-Gal (right) as specified in axis for 7 days (sequential treatment). **(F)** Numerical heat-map representation of normalised mean clonogenic potential after 7 days of CDDP treatment followed by 7 days of senotherapy treatment navitoclax (left) or Nav-Gal (right), where 1 = maximum clonogenic potential corresponding to CDDP 0.5 μM condition (n=2). **(G)** Normalised mean clonogenic potential of A549 cells upon increasing concentrations of Navitoclax (left) or Nav-Gal (right) as a single or combined treatment with CDDP. In **(E)** data represent mean ±SEM (n=3), and in **(A, C** and **D)** data represent mean ± SD (n=3), where for each biological repeat the percentage of Annexin V-positive cells was calculated in 3 independent technical repeats per experimental condition. Statistical significance was calculated using two-tailed Student's t-tests; *$p < 0.05$, **$p < 0.001$.

Figure S4. Sequential Nav-Gal treatment after chemotherapy decreases tumour volume and platelet toxicity compared to Navitoclax. (A) A549-xenograft-bearing mice were first treated for a week with cisplatin (CDDP, 1.5 mg/kg three times a week) and then treated daily with Navitoclax (100 mg/kg body weight) or Nav-Gal (85 mg/kg body weight) or their vehicles until end-point. Blood was then collected by cardiac puncture and the platelet count in each group was analysed. **(B)** Tumour volume of A549 xenografts in mice treated as described in **(A)** over time. **(C)** Platelet count in each experimental condition upon end of treatment *in vivo* (vehicle, Navitoclax, Nav-Gal and CDDP + Navitoclax, n=5; CDDP and CDDP + Nav-Gal, n=3). Data represent mean ± SEM. Two-way ANOVA followed by Bonferroni post-tests or one-tailed t-tests were performed to calculate the significance of the results; * $p < 0.05$.

Figure S5. **Concomitant treatment of lung tumours with pro-drug Nav-Gal and cisplatin significantly decreases tumour burden in an orthotopic lung cancer mouse model. (A)** Murine KP lung cancer cells were orthotopically transplanted in the lungs of C57BL/6J mice via tail-vein injection. After 5 days, animals were imaged and randomised into different groups following luciferase signal analysis, and subsequently treated with either vehicle (n=4), cisplatin (1 mg/kg body weight; n=5) or cisplatin and Nav-Gal (85 mg/kg body weight; n=6) as shown in the schematic representation. Mice

were imaged at day 10 and 15 post-transplantation and lungs were collected at end-point for histological analysis. **(B)** Representative images of luciferase activity signal at day 5 (start of treatment) and 15 (end of treatment) of each experimental group are shown. **(C)** Fold-change of relative luciferase activity of each group over-time. **(D)** Representative histological images of lung sections of each experimental group stained for SA--gal activity (in blue), p21 (red), TUNEL (green) and ki67 (red). Data in **(C)** represent mean ± SEM, and statistical significance was calculated by one-way ANOVA followed by Bonferroni post-tests.

Figure S6. Gating strategy for the analysis of Annexin V-positive platelets (apoptotic platelets) in samples from **(A)** human and **(B)** mouse blood. Total platelet events were gated based on scatter signals (SSC-A vs FSC-A) and then free platelets were gated through CD41 expression. The percentage of apoptotic platelets was determined by analysing the Annexin V-positive population.
